# A genomic analysis of the archaeal system *Ignicoccus hospitalis-Nanoarchaeum equitans*

**DOI:** 10.1186/gb-2008-9-11-r158

**Published:** 2008-11-10

**Authors:** Mircea Podar, Iain Anderson, Kira S Makarova, James G Elkins, Natalia Ivanova, Mark A Wall, Athanasios Lykidis, Kostantinos Mavromatis, Hui Sun, Matthew E Hudson, Wenqiong Chen, Cosmin Deciu, Don Hutchison, Jonathan R Eads, Abraham Anderson, Fillipe Fernandes, Ernest Szeto, Alla Lapidus, Nikos C Kyrpides, Milton H Saier, Paul M Richardson, Reinhard Rachel, Harald Huber, Jonathan A Eisen, Eugene V Koonin, Martin Keller, Karl O Stetter

**Affiliations:** 1 Biosciences Division, Oak Ridge National Laboratory, 1 Bethel Valley Rd, Oak Ridge, TN 37831, USA; 2 DOE Joint Genome Institute, 2800 Mitchell Drive, Walnut Creek, CA 94598, USA; 3 National Center for Biotechnology Information, National Library of Medicine, National Institutes of Health, 8600 Rockville Pike, Bethesda, MD 20894, USA; 4 Verenium Corporation, 4955 Directors Place, San Diego CA 92121, USA; 5 Division of Biological Sciences, University of California San Diego, 9500 Gilman Drive, La Jolla, CA 92037, USA; 6 Lehrstuhl für Mikrobiologie und Archaeenzentrum, Universität Regensburg, Universitätstraße 31, Regensburg, D-93053, Germany; 7 Genome Center, University of California Davis, One Shields Avenue, Davis, CA 95616, USA; 8 Current address: College of Agricultural, Consumer, and Environmental Sciences University of Illinois at Urbana-Champaign, 1101 W Peabody Dr., Urbana, IL 61801, USA; 9 Current address: Biology Department, San Diego State University, 5500 Campanile Drive San Diego, CA 92182, USA; 10 Current address: Amgen Inc., One Amgen Center Drive, Thousand Oaks, CA 91320, USA

## Abstract

**Background:**

The relationship between the hyperthermophiles *Ignicoccus hospitalis *and *Nanoarchaeum equitans *is the only known example of a specific association between two species of Archaea. Little is known about the mechanisms that enable this relationship.

**Results:**

We sequenced the complete genome of *I. hospitalis *and found it to be the smallest among independent, free-living organisms. A comparative genomic reconstruction suggests that the *I. hospitalis *lineage has lost most of the genes associated with a heterotrophic metabolism that is characteristic of most of the Crenarchaeota. A streamlined genome is also suggested by a low frequency of paralogs and fragmentation of many operons. However, this process appears to be partially balanced by lateral gene transfer from archaeal and bacterial sources.

**Conclusions:**

A combination of genomic and cellular features suggests highly efficient adaptation to the low energy yield of sulfur-hydrogen respiration and efficient inorganic carbon and nitrogen assimilation. Evidence of lateral gene exchange between *N. equitans *and *I. hospitalis *indicates that the relationship has impacted both genomes. This association is the simplest symbiotic system known to date and a unique model for studying mechanisms of interspecific relationships at the genomic and metabolic levels.

## Abstract 2

Sequencing of the complete genome of Ignicoccus hospitalis gives insight into its association with another species of Archaea, Nanoarchaeum equitans.

## Background

The crenarchaeaote *Ignicoccus hospitalis *is a specific host for *Nanoarchaeum equitans *in a relationship that is thus far unique, involving two archaeal species [1-3]. *Ignicoccus *species have a chemoautotrophic metabolism that couples CO_2 _fixation with sulfur respiration using molecular hydrogen in high temperature hydrothermal vent systems and thus might resemble organisms that thrived on the primitive, hot and anoxic Earth [4-8]. Uniquely among Archaea, *Ignicoccus *cells are surrounded by two membranes separated by a wide periplasmic space within which vesicles and tubular structures emerge from the cytoplasm [9]. Some of these structures reach and fuse with the outer membrane [10], which has a distinct lipid composition and contains pores of a unique type [11]. The physiological significance of these features and their potential involvement in the relationship with *N. equitans *are unknown.

With a highly reduced genome, *N. equitans *has virtually no obvious metabolic or energetic capabilities and, using unknown mechanisms, must obtain metabolites and energy from *I. hospitalis *by attaching to its surface [3,12,13]. The similarity of the lipid compositions between the cytoplasmic membranes of *I. hospitalis *and *N. equitans *suggests specific lipid partitioning and transport mechanisms [13]. In addition, carbon labeling and cell fractionation have demonstrated the transfer of amino acids from *I. hospitalis *to *N. equitans *[3]. In co-cultures with *I. hospitalis*, *N. equitans *cells can be regularly observed detached and, for some time, they appear to maintain their membrane integrity, at least based on live-dead staining [3]. The mechanism of separation from the host cell and the potential existence of a reattachment process are still unknown. Attempts to propagate *N. equitans *in co-cultures with other archaea, including other species of *Ignicoccus*, have not been successful, suggesting that the relationship with *I. hospitalis *is highly specific and involves a recognition mechanism [3]. While under laboratory conditions the effects exerted by *N. equitans *on its host range from mildly to moderately inhibitory [1,3], *Nanoarchaeum *might confer on *Ignicoccus *an advantage in colonizing hydrothermal vents [14]. As its exact nature remains elusive, provisionally describing this relationship as a symbiosis is compatible with representing either a novel type of interspecific association or fitting within recognized categories of microbial interactions [15].

It has been proposed that genomic characteristics of *N. equitans *such as the numerous split genes and extremely compact genome might be signatures of an ancient lineage [12,16], although a viable alternative seems to be that at least some of these features are secondarily derived [17]. The age of the *Ignicoccus*-*Nanoarchaeum *relationship is unknown, although both organisms represent hyperthermophilic lineages and inhabit types of ecosystems that are often considered to be ancient [7,18]. This system provides insights into physiological mechanisms of interaction between unicellular organisms and can offer clues to evolutionary events that shape the genomes of symbionts leading to physiological interdependence. The *Ignicoccus*-*Nanoarchaeum *relationship might even serve as an analogous model to proposed symbiotic events that could have led to the formation of eukaryotic cells [19]. To advance the study of this relationship at the genomic level, we sequenced the complete genome of *I. hospitalis*, complementing that of *N. equitans *[12]. In this study, in conjunction with the available physiological and morphological data, we performed the genomic analysis and metabolic reconstruction of *I. hospitalis*, as a step to deciphering the evolutionary history and the molecular mechanisms that enable the symbiotic relationship between the two archaea.

## Results and discussion

### A minimal genome

**Figure 1:**
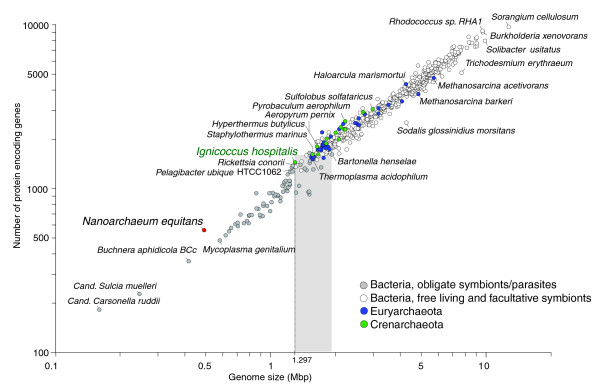
Relationship between the genome size and the number of protein-coding genes in 623 complete archaeal and bacterial genomes, based on data in IMG version 2.5 (March 2008). The line points to *I. hospitalis *having the smallest genome among independently replicating organisms. The genomes of obligate parasites/symbionts are represented by grey circles. The shaded region of genome sizes spans the transition between obligate symbionts/parasites and free-living organisms.

The genome of *I. hospitalis *consists of a single circular chromosome (Table 1). At 1,297,538 bp, the genome of *I. hospitalis *is the smallest among free-living organisms, which do not require a continuous association with another species and can replicate independently (Figure 1). Even the combined gene complement of *I. hospitalis *and *N. equitans *(1,434 and 556 protein-coding genes, respectively) is significantly smaller than that of average free-living bacteria (approximately 3,600 genes) or archaea (approximately 2,300 genes), based on the available completed genomes. The size distribution of 623 complete microbial genomes indicates that the 1-2 Mbp range includes both obligate symbionts/parasites as well as free living bacteria and archaea (Figure 1). The minimal genome for free-living organisms may therefore be on the order of 1 Mbp, several taxonomically and metabolically distant archaeal and bacterial lineages having independently reached near-minimal functional gene sets for their respective ecological niches.

**Table 1:** General features of the *I. hospitalis *genome

Parameter	Value	%
Chromosome size (bp)	1,297,538	
Chromosome G+C content		56.5
Total number of genes	1,494	100
Protein coding genes	.1,444	96.6
RNA genes	.50	3.3
Genes with function prediction	.885	59.2
Genes without function prediction	559	37.4
Genes in ortholog clusters	.1,149	76.9
Genes in paralog clusters	.406	27.2
Fusion genes	.27	1.8
Genes assigned to COGs	.972	65.1
Genes assigned to arCOGs	1,155	80.5
Genes assigned to Pfam domains	.875	58.6
Genes with signal peptides	.213	14.3
Genes with transmembrane helices	.216	14.5
Putative pseudogenes (RNA + proteins)	12	0.8

The sizes of microbial genomes are the result of dynamic equilibria between contraction by deletions and expansion due to duplications, lateral gene transfer and insertion of mobile DNA. For free-living organisms with very large effective population sizes, genome streamlining is likely to be a selective consequence of reducing the metabolic burden to maintain DNA of little adaptive value, as illustrated by the genomes of such highly successful and widespread lineages as *Prochlorococcus *and *Pelagibacter *[20,21]. An alternative (but not necessarily exclusive) hypothesis links genome reduction to elevated mutation rates in large populations. Accumulation of mutations could lead to inactivation and loss of genes that make weak contribution to the fitness of the respective organisms [22]. *Ignicoccus*, however, inhabits heterogeneous, geographically dispersed and relatively ephemeral hydrothermal marine environments. Such organisms generally have small effective populations and experience periodic bottlenecks and limited gene flow [23]. Conceivably, in a case like this, genome contraction might have to do with the very active recombination and DNA repair that organisms inhabiting extreme environments employ for maintaining genomic integrity. Frequent recombination might not only efficiently remove deleterious mutations induced by the environmental conditions but also generate diversity and increase the fixation rate of adaptive alleles [24,25]. A high frequency of illegitimate, intra-chromosome recombination could also be effective in preventing genome expansion by increasing the frequency of deletions and counteracting gene duplication. This might explain the reduced genome size in many members of the Archaea and contribute to their proposed higher adaptability to chronic energy stress [26]. While we expect these general principles to be valid in the *Nanoarchaeum-Ignicoccus *system as well, the co-evolution of these two organisms also left unique imprints on their physiology [2,3]. The most striking effect of this co-evolution, however, is the massive gene loss in *N. equitans*, resembling that of obligate intracellular bacterial symbionts and, as an extreme case, that of eukaryotic organelles [12].

The recently published database of archaeal clusters of orthologous genes (arCOGs) provides a framework for comparing the *I. hospitalis *genomic data to genes from 41 previously sequenced archaeal genomes organized into sets of probable orthologs [27]. Of the 1,434 annotated *I. hospitalis *protein-coding genes, 1,155 (80.5%) were assigned to arCOGs, a coverage that is the lowest among the Desulfurococcales (85% on average) and overall among thermophilic Crenarchaeota.

*I. hospitalis *lacks orthologs of 19 genes from the Crenarchaeota core (that is, genes that are represented in all 12 available genomes of thermophilic species of Crenarchaeota included in the arCOGs) [27] (Table S1 in Additional data file 1). None of these genes include components of information processing systems, indicating that these systems are largely intact in *I. hospitalis *despite the small genome. The missing genes encode, primarily, diverse metabolic enzymes, some of which - for example, thymidylate kinase - catalyze essential reactions. Conceivably, these enzymes are substituted for by distant homologs that so far remain undetected or by analogs.

**Figure 2:**
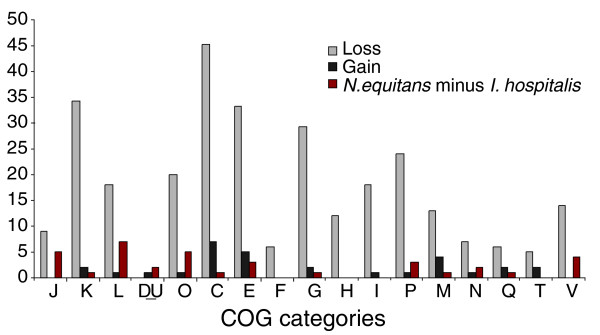
Numbers of arCOGs in different functional categories (COG classification) lost or gained in the *I. hospitalis *lineage. The sets of lost and gained genes were derived on the basis of a comparison of the *I. hospitalis *gene compliment with the reconstructed gene set of the last common ancestor of Desulfurococcales [27] (see Additional data files). The numbers of arCOGs in each category that are present in *N. equitans *but are absent in *I. hospitalis *are also indicated. The one letter code for COG categories is the following: amino acid transport and metabolism (E); carbohydrate transport and metabolism (G); cell cycle control, cell division, chromosome partitioning (D); cell motility (N); cell wall/membrane/envelope biogenesis (M); coenzyme transport and metabolism (H); defense mechanisms (V); energy production and conversion (C); inorganic ion transport and metabolism (P); intracellular trafficking, secretion, and vesicular transport (U); lipid transport and metabolism (I); nucleotide transport and metabolism (F); posttranslational modification, protein turnover, chaperones (O); replication, recombination and repair (L); secondary metabolites biosynthesis, transport and catabolism (Q); signal transduction mechanisms (T); transcription (K); and translation, ribosomal structure and biogenesis (J).

Using the assignment of *I. hospitalis *genes to arCOGs, we applied weighted parsimony to perform a reconstruction of gene gain and loss events in archaea [27,28], with an emphasis on the *I. hospitalis *lineage. The small genome size appears to be a result of gene loss that has vastly predominated the evolution of this lineage: it was inferred that approximately 484 arCOGs were lost, compared to the inferred gain of only 56. Approximately 946 arCOGs (1,094 genes, representing 76% of the *I. hospitalis *gene set) appear to have been inherited from the last common ancestor of the Desulforococcales, the order to which *Ignicoccus *belongs, together with *Aeropyrum pernix, Hyperthermus butylicus *and *Staphylothermus marinus*. The functional distribution of the lost genes is consistent with the fact that *I. hospitalis *is an obligate anaerobic autotroph. In contrast to *A. pernix*, numerous genes related to aerobic metabolism as well as catabolism and transport of amino acids, sugar and nucleotides were lost, along with many transcriptional regulators (Figure 2; Table S2 in Additional data file 1). An analysis of arCOGs that are present in *N. equitans *but absent in *I. hospitalis *does not suggest that the inferred gene loss in *I. hospitalis *was accompanied by transfer of potentially essential functions to the symbiont (Table S3 in Additional data file 1). *Ignicoccus *is far removed from the root of the tree of thermophilic Crenarchaeota (whether the tree is constructed for rRNA or various informational proteins), and the tree, including basal branches, is dominated by heterotrophs and mixotrophs (Figure S1 in Additional data file 2). Thus, the alternative scenario, namely, that *Ignicoccus *reflects the ancestral state for this entire group, is not supported by the phylogenetic analyses. However, this might reflect our incomplete sampling of the archaeal diversity and the bias towards isolation and characterization of heterotrophs. A better understanding of the direction of evolution in archaeal genome size and architecture will require a significant increase in the number and diversity of cultivated species and sequenced genomes, including close relatives of *I. hospitalis *and additional chemolithoautotrophs.

**Figure 3:**
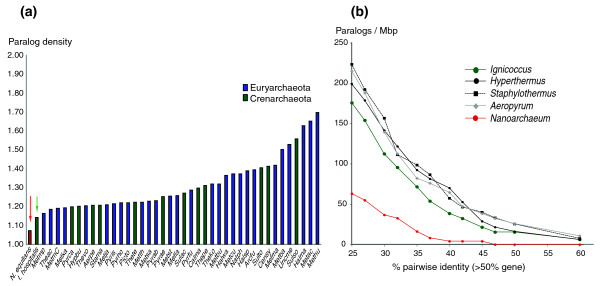
Paralog distribution in completely sequenced archaeal genomes. **(a) **The average number of paralogs in arCOGs for completely sequenced archaeal genomes. The arrows point to the vales for *N. equitans *and *I. hospitalis *genomes, respectively. **(b) **Paralog density in completed genomes of species from the order Desulfurococcales and in *N. equitans*, determined by blastclust using a variable identity threshold over at least 50% of the aligned pairs of sequences.

The reduced frequency of duplicated genes (paralogs) in *I. hospitalis *compared to all other archaea except *N. equitans *(Figure 3) and the absence of transposable elements support the hypothesis of genome streamlining. Furthermore, approximately 180 chromosomal gene clusters that are typically conserved in archaea are disrupted in the genome, including some of the ribosomal operons as well as those encoding the proteasome components, ATP synthase and DNA topoisomerase VI. As it is unlikely that so many gene clusters and operons have independently assembled in archaeal lineages not directly related, the architecture of the *I. hospitalis *genome suggests that recombination events have resulted in gene cluster fragmentation, deletions, and may have restricted gene family expansion. On the other hand, it is notable that several families of paralogous genes are uniquely expanded in *I. hospitalis *(Table S4 in Additional data file 1). The most intriguing is the presence of 10 genes that encode WD40-repeat-containing proteins. Proteins containing WD40 repeats are among the most abundant and highly conserved in eukaryotes, where they are key structural components of a variety of macromolecular complexes [29]. Proteins containing these repeats are also widely scattered among archaea and bacteria but are mostly encoded in (relatively) large genomes [30]. In particular, among archaea, we have detected comparable expansions of WD40-containing proteins only in Methanosarcinales, a group of Euryarchaeota that displays significant gene gain [27]. Conceivably, the WD40-proteins of *I. hospitalis *are involved in the organization of specific protein complexes and/or cellular compartments, and potentially might contribute to the interaction with *N. equitans*. Similarly, *I. hospitalis *encodes 9 proteins containing the V4R domain and 12 proteins containing the CBS domain, both small-molecule-binding domains that are likely to be involved in metabolic regulation and signaling [31,32] (Table S4 in Additional data file 1). Considering the homology identified between the V4R domain and a component of the eukaryotic Golgi vesicle transport machinery [33], some of the expanded V4R gene family members also might be implicated in the unique vesicle formation process that has been observed in *Ignicoccus *[9].

**Figure 4:**
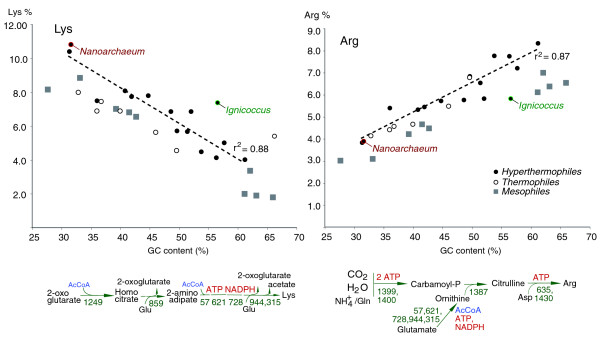
Lysine and arginine use in archaeal proteomes, relative to genome G+C content. The dotted lines represent the linear fit to the hyperthermophile data and the goodness of fit values. The archaeal classification as hyperthermophiles, thermophiles and mesophiles follows that of the NCBI Genome Project database [100]. The proposed pathways for the biosynthesis of the two amino acids, the genes predicted to be involved and the metabolic costs of the two reactions are shown below the graphs.

In addition to streamlining, selection for reducing metabolic cost in *I. hospitalis *may have impacted its proteome composition. In hyperthermophiles, certain biases in amino acid usage have been associated with side chain physical and chemical properties that contribute to increased protein stability [34,35]. For example, a preference for lysine over arginine has been attributed to a greater flexibility of the lysine side chain, which entropically stabilizes the folded state of proteins [36]. While the overall amino acid usage in the *N. equitans*-*I. hospitalis *proteomes follows the distribution observed for other hyperthemophiles, there is a significant increase in lysine over arginine usage in *I. hospitalis *relative to the values that could be predicted from the GC content (Figure 4; note that the two positively charged amino acids, lysine and arginine, are often interchangeable in proteins but are encoded by contrasting codons, namely AAA/G for lysine, and CGX and AGA/G for arginine, hence the strong correlation of the abundance of these amino acids with the GC content). This discrepancy could be explained by selection at the genomic level against using the metabolically more expensive arginine. Arginine biosynthesis in *Ignicoccus *is predicted to proceed via carbamoyl-phosphate and would require five ATP equivalents, whereas lysine, synthesized from 2-oxoglutarate via the aminoadipate pathway, would use two ATP equivalents (Figure 4). Metabolic cost and nutrient availability have been proposed to play a selective role in the evolution of genome size, GC content and amino acid use in organisms that inhabit oligotrophic or energetically poor environments [20,26,37]. Since sulfur-hydrogen respiration is energetically weak [38], such genomic and proteomic adaptations allow *I. hospitalis *not only to be a competitive vent colonizer but also to support *N. equitans*. At present, in the absence of sequence data from other species of *Ignicoccus*, we cannot distinguish if the relationship with *N. equitans *has directly influenced these genomic features of *I. hospitalis*.

### Lateral gene transfer

The cell-cell contact between *I. hospitalis *and *N. equitans *seems to present an opportunity for extensive lateral gene transfer (LGT). LGT is considered to play a major role in microbial genome evolution and is well-documented in symbiotic systems and in environmental microbial communities [39-42]. Recent LGT events are readily detected with various methods based on nucleotide composition or codon usage, but methods that rely on protein sequence similarity and phylogenetic trees are more informative for ancient LGT events [43]. To analyze the *I. hospitalis *genome for potential LGT events, we therefore combined automatic genome-wide phylogenetic reconstruction using PyPhy [44] with similarity searches and COG distribution analysis. The LGT candidates were further analyzed using hand-curated alignments and maximum likelihood phylogenetic analyses. Identifying the LGT direction requires analysis of conflicts between the topologies of the corresponding gene trees and the adopted species tree. The position of *N. equitans *within the Archaea is controversial and ranges from representing a distinct and basal phylum [1,12,16] to being a derived member of order Thermococcales from the Euryarchaeota [17]. Many gene trees identify the Thermococcales as an early diverging lineage, which further complicates this distinction. *Ignicoccus *on the other hand has been confidently assigned to order Desulfurococcales from the Crenarchaeota based on phylogenetic and arCOG analysis. Therefore, when attempting to infer direction of LGT, we relied on the phylogenetic placing of *N. equitans *and *I. hospitalis *genes relative to other crenarchaeal homologues, especially those from the Desulfurococcales (*Aeropyrum*, *Hyperthermus *and *Staphylothermus*).

**Figure 5:**
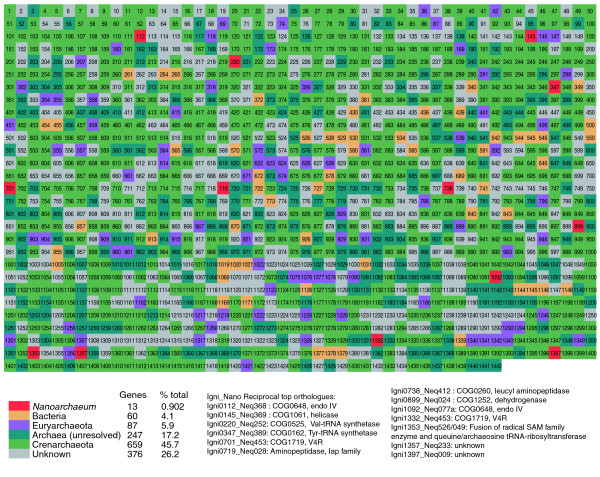
Taxonomic classification of *I. hospitalis *protein-coding genes based on phylogenetic and COG distribution analyses. Genes labeled in green or blue-green are of Crenarchaeota-type or are of unresolved archaeal nature, respectively. Genes that could represent horizontal gene transfers from Euryarchaeota or Bacteria are labeled in purple and yellow, respectively. Genes that have their closest ortholog in *N. equitans *are labeled red and are described in the table. Genes labeled in gray lack recognizable homologues in other microbial genomes or have unresolved phylogenies preventing confident affiliation to either Archaea or Bacteria.

A small fraction of *I. hospitalis *genes (approximately 6%) appear to have been transferred from lineages within Euryarchaeota, while approximately 4% seem to be of bacterial origin (Figure 5). Many of those genes encode subunits of protein complexes involved in energy metabolism or transporters and might have been acquired by *I. hospitalis *as small clusters or operons. Examples of putative 'bacterial' gene clusters include those encoding bacterial type polysulfide reductase (Igni528-530), the multisubunit putative Ech hydrogenase (Igni542-546, Igni1144-148) and a nitrate reductase-like complex (Igni1377-1379). Among the clusters of apparent origin from Euryarchaeota are genes encoding ABC-type transporters for antibiotics and molybdate (Igni146-147, Igni1340-1343) as well as a 2-oxoacid:ferredoxin oxidoreductase complex (Igni1075-1078). Other genes encoding characteristic proteins of Euryarchaeota are scattered in the genome (for example, the CrcB-like integral membrane protein Igni921, a 6Fe-6S prismane cluster-containing protein Igni960, micrococcal thermonuclease Igni1343, thermophilic glucose-6-phosphate isomerase Igni415). If *N. equitans *is a derived member of Thermococcales, as some gene trees and genomic analyses suggest [17,27], then some of the putative euryarchaeal LGTs in the *I. hospitalis *genome might actually represent transfers from *N. equitans*. Such transfers could have occurred during extensive genome degradation suffered by *N. equitans *associated with elimination of metabolic functions, similar to cases of nuclear transfer of symbiont genes during eukaryotic organelle formation. Additional LGTs from bacteria and/or archaea, including *N. equitans*, might be hidden in the large number of genes (>600 or approximately 40% of the open reading frames) that either lack detectable homologs or are placed unresolved within the Archaea due to insufficient phylogenetic signal.

**Figure 6:**
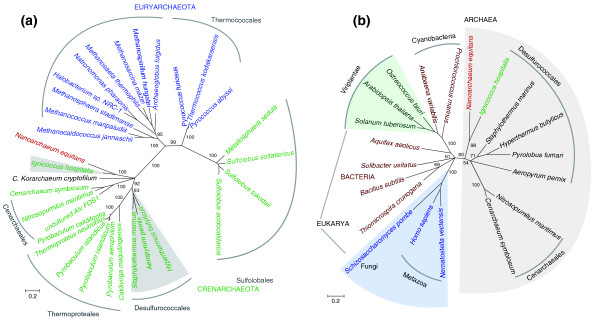
Maximum likelihood phylogenetic trees **(a) **of archaeal valyl-tRNA synthetases and **(b) **of leucyl aminopeptidases representing the three domains of life and including all the known archaeal sequences. Numbers indicate bootstrap support based on 100 replicates. Where the value was <50, the branch was collapsed. The scale bar indicates the inferred number of substitutions per site. The sequence alignments used to generate the trees are provided in the Additional data file 4.

One of the possible outcomes of LGT in symbiotic associations involves orthologous gene displacement in the recipient genome and maintenance of the gene in the donor genome as well. In the *N. equitans-I. hospitalis *system, we identified 13 such cases, in which the orthologs in both genomes are each other's closest homologues (Figure 5). Several of the genes appear to have been transferred from *N. equitans *to *I. hospitalis*, including ones encoding valyl-tRNA synthetase (Igni220-Neq252), tyrosyl-tRNA synthetase (Igni347-Neq389) and a type IV endonuclease (Igni1092-Neq77a) (Figure 6a; Figure S2 in Additional data file 2). Two genes involved in recombination and repair that form a predicted operon in *N. equitans *(an AP endonuclease 2 family and a DEAD/DEAH box helicase, NEQ368-369) have also been transferred to *I. hospitali*s, either as independent events or becoming separated later by genomic rearrangement (Igni0112 and 0145). Genes encoding aminoacyl tRNA synthetases and recombination and repair proteins are frequently exchanged in microbial communities and might increase the fitness of recipient organisms, for example, by conferring antibiotic resistance in the case of aminoacyl-tRNA synthetases [45,46].

A similar case of lateral transfer likely involved the gene encoding leucyl aminopeptidase (LAP), Igni738-Neq412 (Figure 6b). LAPs are ubiquitous in bacteria and eukaryotes but their presence in archaea is so far strictly limited to the Desulfurococcales and the Cenarchaeales. While no specific function has been described so far for archaeal LAPs, in bacteria they are multifunctional proteins, with roles in protein turnover as well as in transcription control and recombination [47]. The absence of LAP in Euryarchaeota, in *Korarchaeum cryptofilum *(a potentially basal archaeal lineage with affinities with the Crenarchaeota [48]) as well as in two of the four Crenarchaeota orders for which genomic data are available may suggest that the Desulfurococcales and Cenarchaeales acquired the gene via LGT from bacteria. The phylogenetic analysis places the *I. hospitalis *gene close to that of *N. equitans *but not part of the Desulfurococcales clade. The high level of sequence similarity between the *N. equitans *and *I. hospitalis *LAP genes (40%) surpasses that between any of the other Desulfurococcales (approximately 30%). However, the direction of the transfer is uncertain. The exclusion of the *I. hospitalis *LAP from the clade formed by the other Crenarchaeota homologs suggests that the *Ignicoccus *gene may have been acquired from *N. equitans *followed by orthologous gene displacement. Based on this scenario, the original presence of LAP in *N. equitans *would be at odds with its purported affiliation with the Euryarchaeaota and specifically the Thermococcales, which are lacking leucyl aminopeptidases. The alternative hypothesis, transfer of the LAP gene from *I. hospitalis *to *N. equitans*, is challenged by the separation of the *Ignicoccus-Nanoarchaeum *clade from the other Desulfurococcales. Complete genome sequences of other *Ignicoccus *or Nanoarchaeota species may help distinguish between these competing hypotheses.

Genetic information processing in *I. hospitalis*, as inferred from the genome sequence, is typical of the Crenarchaeota. Orthologs of two family B DNA polymerases are present in the genome (Igni62, 690); one corresponds to the aphidicolin-resistant DNA polymerase I (polA), and the other to the aphidicolin-sensitive DNA polymerase II (polB) of *Aeropyrum pernix *[49]. No orthologs of the third family B DNA polymerase or Euryarchaeota-type heterodimeric DNA polymerase were found. Unlike other archaeal genomes, the genes coding for replication initiation/origin recognition factor (Orc1/Cdc6) are not co-localized with the predicted origin of replication [50,51], a characteristic potentially related to general operon fragmentation in *I. hospitalis*. Unlike other archaea, including *I. hospitalis*, that possess DNA primases consisting of a small (catalytic) and large (structural) subunits, *N. equitans *seems to encode a single-subunit primase (NEQ395) in which the small subunit is fused to the carboxy-terminal domain of the large subunit [52] (EVK, unpublished observations). This may be the result of extreme genome contraction in this organism, possibly linked to its symbiotic lifestyle. Similarly, an important molecular machine absent in *N. equitans *but present in *I. hospitalis *is the RNase P complex (RNA and four separate proteins subunits, rpp14, 21, 29 and 30). It has been recently shown that tRNA processing in *N. equitans *is RNase P-independent, most likely because genome shrinkage led to the evolution of leaderless tRNAs that was followed by the loss of all five RNAse P complex genes [53].

### Transport processes

**Figure 7:**
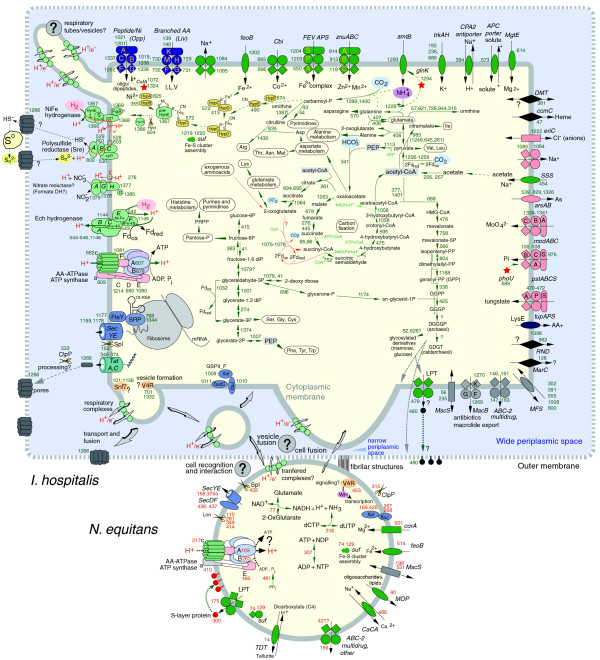
Predicted functional systems and metabolic pathways of the *I. hospitalis-N. equitans *system. The numbers refer to the corresponding genes in the *I. hospitalis *and *N. equitans *genome (green and red, respectively). Some of the biochemical pathways (carbon fixation, amino acid biosynthesis and sugar metabolism) have been experimentally validated [66,69]. Specific subcellular compartments and structures (periplasmic space, vesicles, tubules, pores, fibers) [9,11,62] are indicated and speculative functions are indicated with question marks. Scissors indicate proteases. Stars indicate specific regulatory proteins. Different transporter categories and their individual subunits are indicated by shape symbols and the direction of transport of specific substrates across the membrane is shown by arrows.

The membrane composition of hyperthermophiles is specifically adapted to reduce proton and ion permeability, which increase with temperature [54]. Cyclic tetraether-type lipids (caldarchaeol) that are present in the cytoplasmic membrane of *I. hospitalis *and in the cell membrane of *N. equitans *are especially associated with low permeability [13]. In contrast, the absence of caldarchaeol in the outer membrane of *Ignicoccus *and the presence of protein pores [11] indicate potentially less restrictive exchanges with the environment through the outer membrane. With only eight types of transporters, almost all predicted to be specific for inorganic ions or export of intracellular solutes (Figure 7), *N. equitans *is unlikely to import by itself all of the required metabolic precursors from its host. Consistent with its streamlined genome and autotrophic lifestyle, *I. hospitalis *also encodes very few transporters (<3% of its proteome), the lowest number among the sequenced species of Crenarchaeota. The types of transporters and their inferred specificities are described in Figure 7. A number of inferred subunits of ABC transporters were found in membrane preparations of *I. hospitalis *cells, showing that these proteins are expressed in significant amounts [55]. An unexpected finding for an obligate autotroph was the presence of genes encoding two ABC transporters for oligopeptides and branched amino acids. Under laboratory conditions, it was indeed found that addition of peptides improved growth of *I. hospitalis *[2], suggesting that, in its natural environment, this organism might be opportunistic in utilizing such resources. The different lipid and protein compositions between the cytoplasmic membrane and the outer membrane of *I. hospitalis *[10,13] suggest the existence of specific partitioning mechanisms. The genome encodes a predicted gene (Igni479) from the LolE permease family, an ATP-dependent transport system involved in lipoprotein release that has been shown in *Buchnera *to transport lipids targeted to the outer membrane across the inner membrane [56] and that might play a role in *I. hospitalis *membrane synthesis.

While some proteins may spontaneously insert in the membrane, most transport into and across the membrane requires the function of specialized cellular systems [57]. All the components of the Sec pathway were identified in the *I. hospitalis *genome, including the 7S RNA gene component of the signal recognition particle (Figure 7). Even though potentially functional protein secretion complexes, including the euryarchaeal-specific SecDF are encoded in its genome, *N. equitans *lacks an identifiable 7S RNA gene. Since that component is critical for the assembly of a functional signal recognition particle, it might be synthesized as two separate transcripts, such as some of the tRNAs [58], or might be imported from the host. The Tat system, which transports folded protein across the membrane, is present in *I. hospitalis *but absent in *N. equitans*. For all proteins that are targeted for translocation, the signal peptide has to be removed either during or after translocation. The protease that removes some of the signal peptides in archaea, signal peptidase I, was identified in both genomes (Igni153 and Neq432). *I. hospitalis *also encodes a type IV prepillin peptidase (PibD, MEROPS family A24A, Igni1405), which processes membrane and secreted proteins with a class III signal peptide, including proteins involved in motility (flagellin) and pili formation [59]. Neither *I. hospitalis *nor *N. equitans *appear to have flagellins, although several genes potentially associated with archaeal flagellar or pili assemblies were identified in both genomes (*flaI, flaJ*). While the cells do not appear to be motile, certain appendages and pili-like structures have been observed in electron micrographs [60-62] and might play a role in the interaction between the two organisms.

### Central metabolism

*I. hospitalis *is the first archaeon with sulfur-based autotrophy for which a complete genome sequence is hereby reported. Metabolic reconstruction (Figure 7) points to simple and efficient strategies that fit a streamlined genome. Nitrogen assimilation is predicted to rely on readily available ammonia, the most economical strategy in reduced environments [63]. Ammonia could be acquired through an AmtB transporter (Igni1293), which is apparently co-transcribed with the gene for the nitrogen regulatory protein PII (*glnK*, Igni1294). These genes are widely present in bacteria and most members of the Euryarchaeota but are nearly absent from Crenarchaeota, and probably have been laterally transferred to *I. hospitalis *from a euryarchaeon (Figure 5). GlnK controls the transport of ammonium ions by interacting with AmtB and also activates a type of glutamine synthase (GS) that fixes the ammonia into glutamine. GS is present in all sequenced archaea with the exception of *N. equitans *and, by assimilating ammonia into the amide group of L-glutamine, makes it available to downstream glutamine-dependent amidotransferases. One such enzyme is glutamate synthase (GltS, Igni408), which is predicted to catalyze the reductive transfer of the amide group from glutamine to 2-oxoglutarate, resulting in glutamate and an amino group donor for transamination reactions. The domain architecture of GltS in *I. hospitalis *is unique and contains a GXGXG structural domain [64] followed by a ferredoxin (4Fe-4S) and a glutamine synthetase FMN-binding domain. The glutamate synthase domain I (GlxB domain) is expressed as a separate polypeptide (Igni407) and it is unclear if these two proteins actually assemble to form glutamine synthase or if GlxB functions independently as a type II glutamine aminotransferase. The two genes are likely cotranscribed with an aspartate/aromatic aminotransferase (Igni406), suggesting a tight coupling of the transamination processes. The GS/GltS operates very efficiently at low concentrations of ammonia and substitutes for the alternative ammonia incorporation mechanisms that use glutamate dehydrogenase (GDH) [63]. GDH catalyzes the reversible conversion of glutamate to 2-oxoglutarate and represents an alternative route for both deamination and ammonium incorporation. It has been suggested that GDH provides the major nitrogen assimilation mechanism in most hyperthermophiles [65], but this is clearly not the case with *Ignicoccus*. This is likely due to the absence of a steady source of exogenous amino acids; thus, the cells must rely on free ammonia, present at concentrations too low for GDH to operate at. However, in *N. equitans*, which lacks the ammonium transporter or a GS/GtlS enzyme pair, limited nitrogen metabolism could rely on a GDH (NEQ077), likely using glutamate imported from the host. The transfer of glutamate from *I. hospitalis *to *N. equitans *has been detected experimentally [3]. It is not clear why *N. equitans *has retained a GDH gene among its very few encoding metabolic enzymes. One possibility could be that GDH would contribute to the cell redox potential by oxidative deamination of glutamate.

Pathways for the synthesis of almost all amino acids can be recognized in the *I. hospitalis *genome, with the exception of proline and homocysteine. Some of the enzymatic activities involved in *I. hospitalis *amino acid biosyntheses have been detected experimentally and labeling experiments have been used to reconstruct most the pathways [66]. The genome also encodes the predicted enzymes of purine, pyrimidine, NAD, riboflavin/FAD, pyridoxal and CoA biosynthesis. The mevalonate pathway for the synthesis of the characteristic archaeal membrane archaeol- and caldarchaeol-type lipids appears to be complete (Figure 7), although enzymes involved in some of the steps have not yet been characterized in archaea [67,68].

*I. hospitalis *utilizes a novel and so far unique autotrophic CO_2_fixation pathway, termed the dicarboxylate/4-hydroxybutyrate cycle [69]. The individual steps of the pathway have been investigated experimentally in detail and most have been confirmed biochemically [66,69,70] (Figure 7). Acetate in the form of acetyl-CoA is carboxylated by a pyruvate ferredoxin oxidoreductase enzyme complex (Igni1256-1259) to form pyruvate, which is then converted to phosphoenolpyruvate by pyruvate:water dikinase (Igni1113). The source of acetyl-CoA may be linked to two adjacent genes potentially encoding an acetyl-CoA synthase (Igni256, 257). Normally, that enzyme is encoded as a single polypeptide. The two genes in *I. hospitalis *may encode the enzyme as two subunits requiring post translational assembly or, alternatively, the two open reading frames could indicate a pseudogene. The level of acetate in the environment where *I. hospitalis *has been isolated from is not known, but the genome encodes a putative sodium-acetate symporter (Igni454) and uptake of acetate has been confirmed experimentally [66]. Since *Ignicoccus *can grow in the laboratory in the absence of acetate, the genome might also encode an acetogenesis mechanism using CO_2_. One potential route is direct reduction of CO_2 _to formate using hydrogen, catalyzed by a putative membrane formate dehydrogenase complex. Genes encoding a membrane complex with similarity to both nitrate reductase and formate dehydrogenase were identified as a likely operon acquired from a bacterium (Igni 1377-1380). However, since neither nitrate respiration in *I. hospitalis *cultures [2] nor the biochemical activity of formate dehydrogenase in cell extracts [66] were detected, the cellular function of that complex remains unclear.

The archaeal-type PEP carboxylase [71] catalyzes the second CO_2 _incorporation reaction, which results in the formation of oxaloacetate, an important precursor for amino acid biosynthetic pathways (Figure 7). Reactions catalyzed by malate dehydrogenase, fumarase, succinate dehydrogenase and succinyl CoA-ligase lead to the synthesis of succinyl-CoA. Until recently, the fate of succinyl-CoA was unclear and, as the reactions that would close the cycle were not apparent based on experimental data or genomic information, the mechanism of acetyl-CoA regeneration remained unknown. Huber *et al*. [69] recently discovered that *I. hospitalis *uses a novel strategy to connect, through succinyl-CoA, the partial reductive citric acid cycle with the 4-hydroxybutyrate route of acetyl-CoA regeneration (Figure 7). Based on this finding, the proposed dicarboxylate/4-hydroxybutyrate cycle appears to be energetically less costly than other carbon fixation cycles operating in archaea [69], further supporting the notion that *I. hospitalis *combines a streamlined genome with efficient metabolic strategies.

Phylogenetic analysis of the two *I. hospitalis *gene clusters encoding oxoacid:ferredoxin oxidoreductase complexes indicates that one of them (Igni1256-1259) belongs to the pyruvate:ferredoxin oxidoreductase family and, therefore, is the likely catalyst for acetyl-CoA carboxylation. The other complex (Igni1075-1078) has a close affinity to a family with oxoglutarate specificity with no close homologs in Crenarchaeota (Figure S3 in Additional data file 2), suggesting acquisition by lateral transfer. The functional inference is based on phylogenetic partitioning of archaeal oxoacid:ferredoxin oxidoreductase genes into distinct clades that correspond to enzymes specific for pyruvate, valerate/isovalerate, or 2-oxoglutarate or that have mixed specificity [72-74]. In addition, alignments of the *I. hospitalis *alpha and beta subunit sequences revealed the presence of motifs conserved in archaeal and bacterial enzymes specific for pyruvate (Igni 1258-1259) or 2-oxoglutarate (Igni 1077-1078) [75-77] (Figure S4 in Additional data file 2).

The function of the predicted 2-oxoglutarate:ferredoxin oxidoreductase (OGOR) complex in *I. hospitalis *remains unclear. 2-Oxoglutarate serves as an entry point in glutamate and lysine biosynthesis and is also linked to the biosynthesis of several other amino acids as shown by carbon tracing and inferred from genomic data [66] (Figure 7). In heterotrophic archaea and bacteria, oxoacid:ferredoxin oxidoreductases are involved in amino acid and sugar fermentation reactions that generate reduced ferredoxin and ATP, although OGOR has been ascribed a biosynthetic function, namely, generation of succinyl-CoA from 2-oxoglutarate [78,79]. By contrast, in anaerobic autotrophs, OGOR is a key enzyme in the reverse citrate cycle, where it catalyzes the fixation of CO_2 _on succinyl-CoA with the formation of 2-oxoglutarate [70]. However, this reaction has not been detected in *I. hospitalis *cell extracts and carbon isotope tracing does not support its occurrence in laboratory cultures [66]. In fact, succinyl-CoA produced by the first half of the carbon fixation cycle is reduced by succinyl-CoA reductase to succinic semialdehyde and channeled into the hydroxybutyrate pathway [69]. The same reaction has been shown to connect the 3-hydroxypropionate with the 4-hydroxybutyrate pathways in another recently discovered novel carbon fixation cycle in the crenarchaeaote *Metallosphaera sedula *[80]. However, unlike the succinyl-CoA reductase from *M. sedula*, which uses NADPH as electron donor, the enzyme in *I. hospitalis *requires reduced ferredoxin [69]. A speculative role for OGOR could be to provide a ferredoxin-based electron shuttle between oxoglutarate and succinyl-CoA, at the expense of a fixed carbon (Figure 7). Such coupling has been shown to be important in the anaerobic metabolism based on aromatic compounds in *Thauera aromatica*, OGOR providing benzoyl-CoA reductase with reduced ferredoxin [75]. In *Ignicoccus*, under active growing conditions, such a reaction based on *de novo *synthesized 2-oxoglutarate does not seem advantageous as it would increase the succinyl-CoA pool at the loss of an acetyl-CoA while sufficient reduced ferredoxin may be supplied by a hydrogenase. However, under limited CO_2 _and H_2 _conditions, 2-oxoglutarate from the internal pool or derived from exogenous amino acids and peptides could keep the 4-hydroxybutyrate part of the cycle active and generate acetyl-CoA for maintenance functions. Experimental studies will be needed to test this hypothesis and identify the specificity of the predicted OGOR complex.

### Respiration and energetic metabolism

Under laboratory conditions, the only energy yielding reaction that sustains the metabolism of *I. hospitalis *is the oxidation of molecular hydrogen coupled to the reduction of elemental sulfur. While energetically weak (-6.7 kcal/mol) [38], there are indications that this type of respiration might have been used by ancient microbes of the early Archaean [5]. Details of bioenergetic reactions and the mechanisms for generating the membrane chemiosmotic potential in anaerobic hyperthermophilic archaea are still not well understood. Minimal enzymatic components that are required include a membrane hydrogenase complex, a sulfur reductase and an electron transport chain between them. In *I. hospitalis*, there appear to be two clusters of genes encoding subunits of the sulfur/polysulfide reductase complex. The first such cluster (Igni801-803) contains the catalytic reductase (SreA), a 4Fe-4S ferredoxin (SreB) and the membrane anchoring component NrfD (SreC) with eight transmembrane domains. NrfD is thought to participate in the transfer of electrons from the quinone pool into the terminal components of the Nrf pathway. Elsewhere in the genome, a gene cluster (Igni528-530) that appears to be of bacterial origin contains a different NrfD, a periplasmic FeS ferredoxin, as well as a membrane protein with four putative heme binding sites that may serve in the electron transfer chain through the membrane, possibly binding menaquinone. This gene cluster is also present in the related archaeon *Hyperthermus butylicus *[81], suggesting the possibility that it was transferred between the two archaeal lineages after one of them likely acquired it from a delta proteobacterium. Two types of reductase complexes might therefore assemble in *I. hospitalis*, archaeal and bacterial. In other sulfur reducers a periplasmic polysulfide-sulfur transferase (a member of the rhodanese family) facilitates the transfer of low concentrations of polysulfide to the reductase. *I. hospitalis *is the only crenarchaeote that is missing a rhodanese family gene. This could be a result of growing under relatively neutral pH, where polysulfide concentrations may be high enough. Therefore, access of polysulfide to the cytoplasmic membrane, where the reductase complex is likely located, could occur by diffusion across the large periplasmic space after passage though the outer membrane pores.

*Ignicoccus *depends on molecular hydrogen as the sole electron donor. A single predicted operon contains the genes encoding the large and small subunits of a hydrogen uptake NiFe hydrogenase, including the large and small subunits (Igni1366-1369). The heterodimer is exported to the periplasm through the twin-arginine translocation (TAT) system and is assembled with a 4Fe-4S ferredoxin and a membrane protein anchor containing histidine residues that might bind a b-type heme [82]. The formation of the metal-containing active site and the assembly of the hydrogenase is a complex process requiring multiple accessory proteins [83], all of which appear to be encoded in the *I. hospitalis *genome (Figure 7). Hydrogen oxidation is coupled with electron transfer through FeS centers and a putative membrane cytochrome to the quinone pool of the respiratory chain, which contributes to the generation of a membrane potential that drives ATP synthesis. The quinone appears to be associated with the membrane component of the hydrogenase and that of polysulfide reductase, with the exchange of the electrons likely involving formation of respiratory 'supracomplexes' [84]. A separate 'energy-converting' Ni-Fe hydrogenase family complex (Ech), which is evolutionarily related to the energy-conserving NADH:quinone oxidoreductase (complex 1), appears to be encoded by genes in two clusters (Igni542-546 and Igni1144-1146). This hydrogenase is the likely catalyst in maintaining the pool of reduced ferredoxin.

The *I. hospitalis *genome also contains a four gene putative operon with close homologues among the bacterial respiratory periplasmic nitrate reductases (Igni1377-1380). Similarity to formate dehydrogenases was also detected, so the function of the complex is not clear, as nitrate cannot serve as a sole electron acceptor in *Ignicoccus *[2,60]. In bacteria, depending on the composition of the complex, periplasmic nitrate reduction can either contribute to the generation of the proton gradient or serve as an electron sink, eliminating excess reducing equivalents from the cytoplasm [85].

A complete membrane A-type ATPase is predicted to be encoded in the genome of *I. hospitalis*, in contrast with only a subset of subunits in *N. equitans *[12]. While *N. equitans *might be unable to synthesize ATP, the presence of a predicted nucleoside diphosphate kinase (Neq307) suggests that regeneration of the NDP pool is feasible, which might reduce its host dependency by recycling (Figure 7). Since it has few ion transporters and no genes encoding membrane hydrogenases or oxidoreductases, it is unknown if *N. equitans *can independently maintain a membrane potential or whether it needs to acquire such capabilities from its host.

As an obligate anaerobe, *I. hospitalis *requires a mechanism to deal with the toxicity of reactive oxygen species. A superoxide reductase is present (Igni1348) and could detoxify superoxide resulting from oxygen reduction by transition metals. According to a recently proposed mechanism [86], a ferrocyanide complex bound within the superoxide reductase active site may scavenge the superoxide by one-electron redox chemistry while the superoxide reductase iron site remains reduced. The resulting peroxide could be transferred to soluble organic compounds, resulting in the formation of alkyl peroxides that can be reduced by peroxiredoxin. A gene encoding a member of this family is encoded in the genome (Igni459) and a recent proteomic analysis of *I. hospitalis *in laboratory cultures has shown that its product is an abundant cytosolic protein [55].

### Potential molecular and structural determinants of the *I. hospitalis-N. equitans *interaction

Although the recognition and exchange mechanisms between *I. hospitalis *and *N. equitans *remain elusive, the available genomic and ultra-structural data suggest some possible ways of interaction between the two organisms. Since the transporters in both species are few and provide limited specificities, they are unlikely to comprise the main route of metabolite acquisition by *N. equitans*. Similarly, transfer of protein complexes to *N. equitans *from the host by secretion, especially for membrane components, would violate topological and signal sequence constraints of the translocation machinery. Potential vehicles for the transport of metabolites and proteins from *I. hospitalis *to *N. equitans *appear to be the large and variably shaped vesicles and tubes that emerge from the host's cytoplasm [9,10]. Such structures could provide transient or even constant contact between the two cytoplasms once the physical contact between the cells has been established, possibly fulfilling the metabolic and energetic requirements of *N. equitans*. This would also allow it to carry out limited respiration, transport and ATP synthesis and may explain how detached *N. equitans *cells or cells not in direct contact with the host can survive for some time. Electron microscopy studies have indicated that some of the *I. hospitalis *periplasmic vesicles fuse with the outer membrane, which likely results in their contents being released into the environment [9,10]. This release of small molecules and, perhaps, peptides might provide chemical cues to *N. equitans *for host recognition and attachment. Since neither of the two organisms appears to be motile, the actual mechanism by which they find each other and become attached in the turbulent hydrothermal vent environment remains enigmatic.

Recent ultra-structural and physiological studies have shown that a physical connection can form between the two organisms [3,62]. Three-dimensional reconstructions point to a dynamic type of interaction, some *N. equitans *cells contacting the outer membrane of *I. hospitalis *in places where the host periplasmic space is wide and contains cytoplasmic vesicles while others are attached to regions with a very narrow periplasm and displaying fibrilar structures [62]. The steps and molecular determinants of the cell-cell recognition and interaction and the membrane and periplasm dynamics remain uncharacterized. The cytoplasmic membrane of *Ignicoccus *itself is highly 'corrugated', as shown in sections and three-dimensional reconstructions, thereby increasing its surface significantly; in addition, it spontaneously evaginates in the absence of *N. equitans *[2,9,10,62]. Therefore, the physiological role of the conglomerate of tubes and vesicles and the significance of the wide periplasmic space probably extends beyond their possible connection to *N. equitans*. As energy generation resides at the level of the cytoplasmic membrane, these structures could provide a substantially increased respiratory surface confined in the space surrounded by the outer membrane, analogous to the eukaryotic mitochondrial cristae. Vesicles might concurrently transport specific lipids and proteins to the outer porous membrane, which in this case would serve not only as a protective barrier but also for controlling gas and solute exchange. This could represent a mechanism enabling *Ignicoccus *species to rely exclusively on the low energetic yield of the sulfur-hydrogen respiration to sustain an elevated turnover of cellular components at high temperature. Combined with the obligate CO_2 _autotrophy and efficient metabolism, such adaptations might allow *Ignicoccus *to outcompete heterotrophs in colonizing emerging hydrothermal vent niches that are still poor in dissolved organic compounds.

## Conclusion

The combinations of ecophysiological and morphological features that collectively enable the *I. hospitalis-N. equitans *relationship are encoded within a surprisingly simple genomic blueprint. The genome of *I. hospitalis *is the smallest among free-living bacteria and archaea, shows evidence of gene exchange with *N. equitans *and encodes streamlined biochemical functions necessary for a chemoautotrophic metabolism relying on carbon dioxide, hydrogen and sulfur. Aside from selection pressure against genome expansion in a restrictive environmental niche, the two organisms have coevolved, leading to symbiotic specificity and gene exchange. In addition, *I. hospitalis *appears to have acquired a significant number of genes and predicted operons from Bacteria and Euryarchaeota, some of them encoding membrane-associated complexes involved in transport and energetic metabolism. This unicellular symbiotic system might resemble relationships that gave rise to eukaryotic organelles. The availability of complete genomic data for both organisms opens the possibility to study interspecific gene regulatory networks and identify proteins that might be exchanged between interacting cells.

## Materials and methods

### Genome sequencing and functional annotation

*I. hospitalis *KIN4I cells (DSMZ strain 18386) were grown as described in [2]. DNA was isolated from frozen cells using an alkaline lysis followed by proteinase K digestion method [87]. Sequencing and assembly were performed at the DOE Joint Genome Institute, Walnut Creek, CA, USA using the standard microbial genome sequencing pipeline [88] based on a combination of 3-, 6- and 40-kb (fosmid) DNA libraries. The Phred/Phrap/Consed software package was used to assemble and assess quality [89]. Possible miss-assemblies were corrected and gaps between contigs were closed by editing in Consed, custom primer walks or PCR amplification and sequencing. The estimated error rate in the completed genome sequence of *I. hospitalis *is less than 1 in 50 kbp.

Automated gene prediction was performed by using the output of Critica complemented with the output of Glimmer as part of the genome annotation pipeline at Oak Ridge National Laboratory (ORNL), Oak Ridge, TN, USA. The predicted coding sequences were translated and used to search the National Center for Biotechnology Information (NCBI) nonredundant database, UniProt, TIGRFam, Pfam, PRIAM, KEGG, COG, and InterPro databases. The tRNAScanSE tool [90] was used to find tRNA genes, whereas ribosomal RNAs were found by using BLASTn against the ribosomal RNA databases. The RNA components of the protein secretion complex and the RNaseP were identified by searching the genome for the corresponding Rfam profiles using INFERNAL [91]. Transporter proteins were initially identified based on similarity to transporter categories in GOG and Pfam and were further analyzed using the Transporter Classification Database [92]. Additional gene prediction analysis and manual functional annotation was performed within the Integrated Microbial Genomes (IMG) platform developed by the Joint Genome Institute, Walnut Creek, CA, USA [93]. The complete genome sequence has been deposited in GenBank [GenBank:CP000816.1].

### Comparative genomic analysis

Analysis of the *I. hospitalis *and *N. equitans *genomes were carried out using the IMG system [93]. The genes referred to throughout the text and figures correspond to the assigned open reading frame numbers in the two genomes. Putative operons were identified using the method of Overbeek *et al*. [94]. Structure fold prediction of membrane proteins with no detectable similarity to other database sequences was performed using Phyre [95]. To calculate the frequency of paralogs in the different archaeal genomes, blastclust analyses were performed using the translated coding sequences and varying the similarity threshold for sequence inclusion into clusters. To derive the amino acid usage statistics for archaeal genomes, the percentages of amino acids encoded within each protein were first calculated and used to determine the overall percentage for the whole proteome. The frequency for each amino acid use was then analyzed graphically relative to the GC content of the genome considering that the GC content can influence codon usage. Archaeal COG analyses for *I. hospitalis *and *N. equitans *were performed as described [27]. Analysis of genome sizes of bacteria and archaea was based on genomic data available in IMG (March 2008 version). A table containing the accession numbers for all the genomes as well as all the numerical parameters and classification used in the analysis is provided as Additional data file 3.

### Phylogenetic analysis

To identify the potential presence of laterally transferred genes in *I. hospitalis*, we first used the Pyphy system [44] to automatically calculate individual phylogenetic trees for every gene in the genome. Briefly, each protein sequence was blasted against a local version of a non-redundant protein database (SWISS and TREMBL) and sequences with significant hits (<10e-6) were retrieved and aligned with the query sequence using CLUSTALW. Phylogenetic trees were then constructed using PAUP* with the neighbor joining and parsimony methods with 100 bootstrap replicates. Because the automatic 'phylogenetic connection' calculated by Pyphy and displayed as the phylome map of the genome was at times affected by poor bootstrap support values or unresolved trees, we visually inspected each tree and, when sufficient confidence was present, a broad phylogenetic connection to the Crenarchaeaota, Euryarchaeota or Bacteria was assigned to the *I. hospitalis *gene. For numerous genes, although the *Ignicoccus *gene was clearly of archaeal type, either the phylogenetic signal was insufficient or the evolutionary history of that gene across Archaea involved numerous potential LGTs. Such genes have been generically designated as 'archaeal'. When no close homologues for *I. hospitalis *genes were found or the phylogenetic trees included archaeal and bacterial genes but were not sufficiently resolved, such genes were designated as 'unknown' phylogenetic type. Finally, when the closest hit and the resulting phylogenetic trees indicated a *N. equitans *gene as the closest homologue, those genes were designated as potential LGTs within the *N. equitans-I. hospitalis *system. We also used the arCOG analysis to improve the phylotyping information for some of the functional gene categories that were not resolved by phylogenetic analysis.

Genes representing potential LGTs within the *N. equitans-I. hospitalis *system were subjected to a more extensive phylogenetic analysis. Sequence alignments were obtained using a combination of alternative methods as implemented on the M-Coffee web server [96]. Following manual alignment curation and masking of regions with high variability that could not be confidently aligned, the amino acid substitution model best fit for each gene was chosen using the software Modelgenerator v84 [97]. Maximum likelihood phylogenetic trees were constructed using PhyML v2.4.4 [98] using the parameters identified by Modelgenerator. Alternative tree topologies were also explored using a combination of the software Tree Puzzle and PROML/PHYLIP, as previously described [99]. The protein sequence alignments used to generate the trees for several inferred laterally transferred genes are provided as Additional data file 4.

## Abbreviations

arCOGs: archaeal cluster of orthologous groups; GDH: glutamate dehydrogenase; GS: glutamine synthase; IMG: Integrated Microbial Genomes; LAP: leucyl aminopeptidase; LGT: lateral gene transfer; OGOR: 2-oxoglutarate:ferredoxin oxidoreductase.

## Authors' contributions

MP and KOS conceived and coordinated the study. HS, DH, JRE, ES, AL and PR coordinated and conducted genome sequencing, assembly and sequence data management. MP, IA, KSM, JGE, NI, MEH, MW, AL, KM, WC, AA, NK, MS and EVK performed sequence annotation, comparative genomics and functional inference analyses. MP, KSM, CD and FF performed phylogenetic and phylogenomic analyses. All authors analyzed the results and participated in writing sections of the manuscript. MP assembled and wrote the final version of the manuscript.

## Additional data files

The following additional data are available with the online version of this paper. Additional data file 1 contains a table listing the inferred creanarchaeal core genes lost by *I. hospitalis *(Table S1), a table listing functional categories gained and lost in the *I. hospitalis *genome (Table S2), a table of functional gene categories (arCOGs) present in *N. equitans *but absent in *I. hospitalis *and their distribution in archaeal genomes (Table S3) and a table listing the gene family expansions in the *I. hospitalis *genome (Table S4). Additional data file 2 contains a phylogenetic tree of cultivated thermophilic species of Crenarchaeota based on SSU rRNA sequences (Figure S1), phylogenetic trees of archaeal tyrosyl-tRNA synthetases and of family IV endonucleases (Figure S2), a phylogenetic tree of the alpha subunit of archaeal 2-oxoacid: ferredoxin oxidoreductases (Figure S3) and an amino acid-based sequence alignment of conserved regions of the alpha and beta subunits of pyruvate:ferredoxin oxidoreductases and OGORs (Figure S4). Additional data file 3 contains numerical and classification data associated with all the bacterial and archaeal genomes used in genome size analysis. Additional data file 4 contains the protein sequence alignments used to infer lateral gene transfer of valyl t-RNA synthetase, leucyl aminopeptidase, tyrosyl t-RNA synthetase and endonuclease IV, in phylip format.

## Supplementary Material

Additional data file 1 Table S1: the inferred creanarchaeal core genes lost by *I. hospitalis*. Table S2: functional categories gained and lost in the *I. hospitalis *genome. Table S3: functional gene categories (arCOGs) present in *N. equitans *but absent in *I. hospitalis *and their distribution in archaeal genomes. Table S4: the gene family expansions in the *I. hospitalis *genome. Click here for file

Additional data file 2 Figure S1: a phylogenetic tree of cultivated thermophilic species of Crenarchaeota based on SSU rRNA sequences. Figure S2: phylogenetic trees of archaeal tyrosyl-tRNA synthetases and of family IV endonucleases. Figure S3: a phylogenetic tree of the alpha subunit of archaeal 2-oxoacid: ferredoxin oxidoreductases. Figure S4: amino acid-based sequence alignment of conserved regions of the alpha and beta subunits of pyruvate:ferredoxin oxidoreductases and OGORs. Click here for file

Additional data file 3 Numerical and classification data associated with all the bacterial and archaeal genomes used in genome size analysis. Click here for file

Additional data file 4 Protein sequence alignments used to infer lateral gene transfer of valyl t-RNA synthetase, leucyl aminopeptidase, tyrosyl t-RNA synthetase and endonuclease IV, in phylip format. Click here for file
