# Supplementary material for: A genomic analysis of the archaeal system Ignicoccus hospitalis-Nanoarchaeum equitans
Source: Genome Biol. 2008 Nov 10;9(11):R158. doi: 10.1186/gb-2008-9-11-r158 (PMC2614490; doi:10.1186/gb-2008-9-11-r158)
Supplement: Additional data file 1 — Table S1: the inferred creanarchaeal core genes lost by I. hospitalis. Table S2: functional categories gained and lost in the I. hospitalis genome. Table S3: functional gene categories (arCOGs) present in N. equitans but absent in I. hospitalis and their distribution in archaeal genomes. Table S4: the gene family expansions in the I. hospitalis genome. [file gb-2008-9-11-r158-S1.pdf]

Table S1. Crenarchaeal core genes lost by *I. hospitalis*

COG0160	GabT	E	4-aminobutyrate aminotransferase or related aminotransferase
COG0334	GdhA	E	Glutamate dehydrogenase/leucine dehydrogenase
COG0665	DadA	E	Glycine/D-amino acid oxidase (deaminating)
COG1171	IlvA	E	Threonine dehydratase
COG0503	Apt	F	Adenine/guanine phosphoribosyltransferase or related PRPP-bi protein
COG0125	Tmk	F	Thymidylate kinase
COG0035	Upp	F	Uracil phosphoribosyltransferase
COG1175	UgpA	G	ABC-type sugar transport system, permease component
COG0166	Pgi	G	Glucose-6-phosphate isomerase
COG0469	PykF	G	Pyruvate kinase
COG0524	RbsK	G	Sugar kinase, ribokinase family
COG2084	Mms B	I	3-hydroxyisobutyrate dehydrogenase or related beta-hydroxyac dehydrogenase
COG0330	HflC	O	Membrane protease subunit, stomatin/prohibitin homolog
COG5494	-	O	Predicted thioredoxin/glutaredoxin
COG3371	-	S	Predicted membrane protein
COG1839	-	S	Uncharacterized conserved protein
COG1849	-	S	Uncharacterized protein conserved in archaea
COG1531	-	S	Uncharacterized protein conserved in archaea
COG4755	-	S	Uncharacterized protein conserved in archaea

Table S2. Gain and loss of functional categories in the *I. hospitalis* genome

arCOG	Gene	Type	Annotation	CREN	EURY
Gain					
arCOG01607	PorA	C	Pyruvate:ferredoxin oxidoreductase or related 2-oxoacid:ferredoxin oxidoreductase, alpha subunit	**0**	**0**
arCOG00289	NfnB	C	Nitroreductase	**0**	**8**
arCOG06056	HyaD	C	Ni,Fe-hydrogenase maturation factor	**4**	**0**
arCOG01538	NuoM	C	NADH dehydrogenase subunit M	**0**	**12**
arCOG01495	BisC	C	Molybdopterin oxidoreductase	**4**	**0**
arCOG00959	-	C	Ferredoxin	**0**	**18**
arCOG02430	-	C	6Fe-6S prismane cluster-containing protein	**0**	**11**
arCOG04701	CrcB	D	Integral membrane protein possibly involved in chromosome condensation	**0**	**18**
arCOG02169	-	E	Transglutaminase-like enzyme, putative cysteine protease	**3**	**3**
arCOG01621	Tdh	E	Threonine dehydrogenase or related Zn-dependent dehydrogenase	**0**	**6**
arCOG02305	GlnK	E	Nitrogen regulatory protein PII	**2**	**19**
arCOG02093	LeuA	E	Isopropylmalate/homocitrate/citramalate synthase	**1**	**6**
arCOG00096	GltB	E	Glutamate synthase domain 3	**0**	**15**
arCOG02602	-	G	Thermophilic glucose-6-phosphate isomerase or related metalloenzyme	**0**	**14**
arCOG07813		G	Glycosyl hydrolases family 43	**0**	**3**
arCOG03396	SqhC	I	Squalene cyclase	**0**	**12**
arCOG02643	AcrR	K	Transcriptional regulator, TetR/AcrR family	**0**	**11**
arCOG00876	SSL2	K	DNA or RNA helicase of superfamily II	**6**	**4**
arCOG03192	-	L	Micrococcal nuclease (thermonuclease)	**0**	**14**
arCOG01392	WecB	M	UDP-N-acetylglucosamine 2-epimerase	**1**	**18**
arCOG02080	-	M	S-layer domain	**0**	**10**
arCOG01410	RfaG	M	Glycosyltransferase	**0**	**9**
arCOG01405	RfaG	M	Glycosyltransferase	**0**	**4**
arCOG00589	flhG	N	Antiactivator of flagellar biosynthesis FleN, an ATPase	**1**	**17**
arCOG02871	DsbG	O	Protein-disulfide isomerase	**4**	**0**
arCOG02790	AslA	P	Arylsulfatase A or related enzyme	**0**	**3**
arCOG04989	SmtA	Q	SAM-dependent methyltransferase	**0**	**11**
arCOG01402	SmtA	Q	SAM-dependent methyltransferase	**1**	**7**
arCOG01317	DhlC	R	Sodium:solute symporter	**0**	**4**
arCOG03683	SPS1	R	Serine/threonine protein kinase fused to HEAT repeat domain	**1**	**9**
arCOG03834	-	R	ProFAR isomerase family protein	**0**	**5**

arCOG07561		R	Predicted regulatory protein containing HTH domain	**0**	**6**
arCOG02411	-	R	Predicted nuclease of the RNAse H fold, HicB family	**0**	**12**
arCOG00472		R	Predicted ATPase	**0**	**9**
arCOG06980		S	vWFA domain containing protein	**3**	**0**
arCOG04477	-	S	Uncharacterized protein conserved in archaea	**5**	**25**
arCOG02197	-	S	Uncharacterized conserved protein	**0**	**23**
arCOG03218	-	S	Uncharacterized conserved protein	**0**	**12**
arCOG03216	-	S	Uncharacterized conserved protein	**5**	**12**
arCOG03508	-	S	Uncharacterized conserved protein	**0**	**6**
arCOG05079	-	S	Uncharacterized conserved protein	**0**	**3**
arCOG01240	-	S	Uncharacterized conserved protein	**4**	**0**
arCOG04849		S	Uncharacterized conserved protein	**0**	**5**
arCOG06454		S	Uncharacterized conserved protein	**0**	**4**
arCOG07626		S	Uncharacterized conserved protein	**1**	**1**
arCOG03724		S	Uncharacterized conserved protein	**7**	**1**
arCOG05510		S	Uncharacterized conserved protein	**3**	**0**
arCOG06057		S	Uncharacterized conserved protein	**3**	**0**
arCOG03648		S	Uncharacterized conserved protein	**4**	**0**
arCOG05680		S	Uncharacterized conserved protein	**5**	**0**
arCOG03733		S	Uncharacterized conserved protein	**6**	**0**
arCOG00621	-	S	Uncharacterized conserved DUF39 domain fused to ferredoxin domain	**0**	**15**
arCOG02087	-	S	Predicted membrane protein	**1**	**6**
arCOG03812	-	S	Predicted membrane protein	**1**	**6**
arCOG04967	-	T	PII-like signaling protein	**0**	**13**
arCOG04941	-	T	ACT domain-containing protein	**4**	**12**
arCOG02958	SBH1	U	Preprotein translocase subunit Sec61beta	**1**	**9**

**arCOG**	**Gene**	**Type**	**Annotation**	**CREN**	**EURY**
**Loss**					
arCOG01697	AcnA	C	Aconitase A	**9**	**12**
arCOG01674	AcyP	C	Acylphosphatase	**11**	**21**
arCOG01167	CoxL	C	Aerobic-type carbon monoxide dehydrogenase, large subunit CoxL/CutL homolog	**9**	**3**
arCOG01926	CoxM	C	Aerobic-type carbon monoxide dehydrogenase, middle subunit CoxM/CutM homolog	**9**	**2**
arCOG01925	CoxS	C	Aerobic-type carbon monoxide dehydrogenase, small subunit CoxS/CutS homolog	**9**	**3**
arCOG00707	-	C	Aldehyde:ferredoxin oxidoreductase	**8**	**7**
arCOG01927	-	C	Carbon monoxide dehydrogenase subunit G, CoxG	**9**	**7**

arCOG04237	GltA	C	Citrate synthase	**9**	**13**
arCOG01721	QcrB	C	Cytochrome b subunit of the bc complex	**8**	**6**
arCOG00447	FixB	C	Electron transfer flavoprotein, alpha subunit	**9**	**7**
arCOG00446	FixA	C	Electron transfer flavoprotein, beta subunit	**9**	**7**
arCOG00961	GlpC	C	Ferredoxin	**7**	**0**
arCOG01984	FixX	C	Ferredoxin-like protein	**9**	**3**
arCOG00334	GlpC	C	Fe-S oxidoreductase	**10**	**5**
arCOG01503	HybA	C	Fe-S-cluster-containing hydrogenase component 1	**9**	**0**
arCOG01749	FumC	C	Fumarase	**7**	**7**
arCOG00024	GlpK	C	Glycerol kinase	**6**	**10**
arCOG01238	CyoB	C	Heme/copper-type cytochrome/quinol oxidase, subunit 1	**7**	**3**
arCOG01237	CyoB	C	Heme/copper-type cytochrome/quinol oxidase, subunit 1 and 3	**6**	**5**
arCOG01235	CyoA	C	Heme/copper-type cytochrome/quinol oxidase, subunit 2	**6**	**5**
arCOG01236	CyoA	C	Heme/copper-type cytochrome/quinol oxidase, subunit 2	**7**	**4**
arCOG01609	-	C	Indolepyruvate ferredoxin oxidoreductase, alpha and beta subunit	**11**	**21**
arCOG01755	LdhA	C	Lactate dehydrogenase or related 2-hydroxyacid dehydrogenase	**6**	**7**
arCOG00853	SfcA	C	Malic enzyme	**10**	**17**
arCOG02009	NhaC	C	Na+/H+ antiporter	**2**	**10**
arCOG04335	GltP	C	Na+/H+-dicarboxylate symporter	**2**	**10**
arCOG01252	PutA	C	NAD-dependent aldehyde dehydrogenase	**10**	**23**
arCOG01551	NuoC	C	NADH dehydrogenase subunit C	**11**	**12**
arCOG01537	NuoM	C	NADH:ubiquinone oxidoreductase subunit 4 (chain M)	**10**	**22**
arCOG00288	NfnB	C	Nitroreductase	**10**	**24**
arCOG05865	PckA	C	Phosphoenolpyruvate carboxykinase (GTP)	**6**	**6**
arCOG01004	-	C	Predicted acetamidase/formamidase	**4**	**7**
arCOG01617	Tas	C	Predicted oxidoreductase (related to aryl-alcohol dehydrogenase)	**8**	**7**
arCOG02700	AceF	C	Pyruvate/2-oxoglutarate dehydrogenase complex, dihydrolipoamide acyltransferase (E2) component (C-terminal fragment)	**3**	**0**
arCOG01068	Lpd	C	Pyruvate/2-oxoglutarate dehydrogenase complex, dihydrolipoamide dehydrogenase (E3) component or related enzyme	**11**	**23**
arCOG01606	PorA	C	Pyruvate:ferredoxin oxidoreductase or related 2-oxoacid:ferredoxin oxidoreductase, alpha subunit and gamma	**11**	**19**
arCOG01604	-	C	Pyruvate:ferredoxin oxidoreductase or related 2-oxoacid:ferredoxin oxidoreductase, gamma and delta subunit	**6**	**1**
arCOG01357	-	C	Radical SAM superfamily enzyme	**11**	**9**
arCOG01720	QcrA	C	Rieske Fe-S protein	**7**	**4**
arCOG01097	-	C	Rubrerythrin	**6**	**19**
arCOG04162	SdhD	C	Succinate dehydrogenase, hydrophobic anchor subunit	**3**	**0**
arCOG02244	SdhC	C	Succinate dehydrogenase/fumarate reductase, cytochrome b subunit	**8**	**7**

arCOG00335	-	C	Uncharacterized conserved protein containing ferredoxin-like domain	**10**	**5**
arCOG00506	FpaA	C	Uncharacterized flavoprotein	**4**	**3**
arCOG04358	FdhD	C	Uncharacterized protein required for formate dehydrogenase activity	**9**	**17**
arCOG00915	GabT	E	4-aminobutyrate aminotransferase or related aminotransferase	**12**	**14**
arCOG01798	HisM	E	ABC-type amino acid transport system, permease component	**7**	**13**
arCOG01799	HisJ	E	ABC-type amino acid transport/signal transduction system, periplasmic component/domain	**6**	**14**
arCOG01533	DdpA	E	ABC-type dipeptide transport system, periplasmic component	**7**	**5**
arCOG00923	GlnQ	E	ABC-type polar amino acid transport system, ATPase component	**7**	**13**
arCOG01035	-	E	Alanine dehydrogenase, mu-crystallin homolog	**10**	**18**
arCOG04779	-	E	Asparaginase	**11**	**11**
arCOG04333	-	E	Aspartate/tyrosine/aromatic aminotransferase	**3**	**0**
arCOG00863	ArcC	E	Carbamate kinase	**9**	**9**
arCOG04080	DppA	E	D-aminopeptidase	**3**	**4**
arCOG04172	DapA	E	Dihydrodipicolinate synthase/N-acetylneuraminate lyase	**10**	**27**
arCOG01646	DAP2	E	Dipeptidyl aminopeptidase/acylaminoacyl-peptidase	**11**	**12**
arCOG04053	Ggt	E	Gamma-glutamyltransferase	**10**	**5**
arCOG00027	GadB	E	Glutamate decarboxylase, L-tyrosine decarboxylase or related PLP-dependent protein	**4**	**24**
arCOG01352	GdhA	E	Glutamate dehydrogenase/leucine dehydrogenase	**12**	**19**
arCOG01303	GcvH	E	Glycine cleavage system H protein (lipoate-binding)	**6**	**10**
arCOG00076	GcvP	E	Glycine cleavage system protein P (pyridoxal-binding), C-terminal domain	**5**	**10**
arCOG00077	GcvP	E	Glycine cleavage system protein P (pyridoxal-binding), N-terminal domain	**5**	**10**
arCOG00756	GcvT	E	Glycine cleavage system T protein (aminomethyltransferase)	**6**	**11**
arCOG00755	DadA	E	Glycine/D-amino acid oxidase (deaminating)	**12**	**15**
arCOG01351	ThrA	E	Homoserine dehydrogenase	**9**	**27**
arCOG02462	-	E	Kynurenine formamidase	**7**	**21**
arCOG01888	AmpS	E	Leucyl aminopeptidase (aminopeptidase T)	**3**	**5**
arCOG01316	PutP	E	Na+/proline symporter	**7**	**24**
arCOG01292	GltD	E	NADPH-dependent glutamate synthase beta chain or related oxidoreductase	**9**	**15**
arCOG01511	HyuA	E	N-methylhydantoinase A/acetone carboxylase, beta subunit	**10**	**14**
arCOG01226	ArgK	E	Putative periplasmic protein kinase ArgK or related GTPase of G3E family	**4**	**12**
arCOG00243	LYS9	E	Saccharopine dehydrogenase or related enzyme	**8**	**5**
arCOG01431	IlvA	E	Threonine dehydratase	**12**	**8**
arCOG01459	Tdh	E	Threonine dehydrogenase or related Zn-dependent dehydrogenase	**10**	**17**
arCOG02165	-	E	Transglutaminase-like enzyme, putative cysteine protease	**4**	**9**
arCOG04247	-	E	Zn-dependent carboxypeptidase	**11**	**9**
arCOG04083	-	E	Zn-dependent dipeptidase, microsomal dipeptidase homolog	**9**	**5**

arCOG00031	Apt	F	Adenine/guanine phosphoribosyltransferase or related PRPP-binding protein	**12**	**7**
arCOG04173	Cdd	F	Cytidine deaminase	**8**	**4**
arCOG03448	FUI1	F	Cytosine/uracil/thiamine/allantoin permease	**4**	**0**
arCOG04276	NrdA	F	Ribonucleotide reductase, alpha subunit	**10**	**19**
arCOG01891	Tmk	F	Thymidylate kinase	**12**	**27**
arCOG04128	Upp	F	Uracil phosphoribosyltransferase	**12**	**10**
arCOG00180	MalK	G	ABC-type sugar transport system, ATPase component	**2**	**3**
arCOG00149	UgpB	G	ABC-type sugar transport system, periplasmic component	**5**	**1**
arCOG00153	UgpB	G	ABC-type sugar transport system, periplasmic component	**5**	**7**
arCOG00157	UgpA	G	ABC-type sugar transport system, permease component	**12**	**10**
arCOG00159	UgpE	G	ABC-type sugar transport system, permease component	**9**	**9**
arCOG03661	AMS1	G	Alpha-mannosidase	**5**	**2**
arCOG05412	BglB	G	Beta-glucosidase/6-phospho-beta-glucosidase/beta-galactosidase	**10**	**7**
arCOG01518	FrvX	G	Cellulase M or related protein	**9**	**23**
arCOG00760	CitE	G	Citrate lyase beta subunit	**5**	**4**
arCOG04044	FbaB	G	DhnA-type fructose-1,6-bisphosphate aldolase or related enzyme	**7**	**24**
arCOG01421	GlgP	G	Glucan phosphorylase	**10**	**13**
arCOG00052	Pgi	G	Glucose-6-phosphate isomerase	**12**	**10**
arCOG01420	GlgA	G	Glycogen synthase	**9**	**7**
arCOG03278	-	G	Glycosyl hydrolase family 57	**10**	**14**
arCOG03284	-	G	Glycosyl hydrolase family 57	**7**	**0**
arCOG00152	MalE	G	Maltose-binding periplasmic protein	**4**	**3**
arCOG00699	NagA	G	N-acetylglucosamine-6-phosphate deacetylase	**5**	**1**
arCOG00581	PrpB	G	PEP phosphonomutase or related enzyme	**9**	**4**
arCOG00272	RhaT	G	permease of the drug/metabolite transporter (DMT) superfamily	**9**	**7**
arCOG00139	ProP	G	permease of the major facilitator superfamily	**3**	**4**
arCOG02682	ProP	G	permease of the major facilitator superfamily	**10**	**5**
arCOG01114	PpsA	G	Phosphoenolpyruvate synthase/pyruvate phosphate dikinase	**3**	**4**
arCOG03659	-	G	Predicted N-acetylglucosamine kinase	**5**	**3**
arCOG02877	CDA1	G	Predicted xylanase/chitin deacetylase	**2**	**7**
arCOG04170	GckA	G	Putative glycerate kinase	**11**	**11**
arCOG04120	PykF	G	Pyruvate kinase	**12**	**21**
arCOG04443	RbcL	G	Ribulose 1,5-bisphosphate carboxylase, large subunit	**3**	**16**
arCOG00016	RbsK	G	Sugar kinase, ribokinase family	**12**	**7**
arCOG07490	Ble	G	Uncharacterized protein, probably involved in trehalose biosynthesis	**3**	**1**
arCOG01940	BirA	H	Biotin-(acetyl-CoA carboxylase) ligase	**6**	**23**

arCOG00489	-	H	cob(I)alamin adenosyltransferase	**10**	**12**
arCOG04274	CbiB	H	Cobalamin biosynthesis protein CobD/CbiB	**9**	**27**
arCOG04338	CobS	H	Cobalamin-5-phosphate synthase	**9**	**27**
arCOG01871	-	H	GTP:adenosylcobinamide-phosphate guanylyltransferase	**9**	**27**
arCOG04139	ApbA	H	Ketopantoate reductase	**8**	**13**
arCOG00660	LipA	H	Lipoate synthase	**5**	**5**
arCOG01939	LplA	H	Lipoate-protein ligase A	**6**	**10**
arCOG03837		H	Lipoate-protein ligase A associated domain	**2**	**8**
arCOG04348	UbiE	H	Methylase involved in ubiquinone/menaquinone biosynthesis	**9**	**2**
arCOG04272	CobT	H	NaMN:DMB phosphoribosyltransferase	**8**	**27**
arCOG00535	ThiS	H	Sulfur transfer protein involved in thiamine biosynthesis	**5**	**21**
arCOG00247	MmsB	I	3-hydroxyisobutyrate dehydrogenase or related beta-hydroxyacid dehydrogenase	**12**	**8**
arCOG01279	PaaJ	I	Acetyl-CoA acetyltransferase	**6**	**1**
arCOG01280	PaaJ	I	Acetyl-CoA acetyltransferase	**6**	**4**
arCOG02705	-	I	Acetyl-CoA carboxylase, carboxyltransferase component	**6**	**9**
arCOG01707	CaiA	I	Acyl-CoA dehydrogenase	**9**	**8**
arCOG04100	CaiA	I	Acyl-CoA dehydrogenase	**4**	**3**
arCOG00773	-	I	Acyl-CoA hydrolase	**10**	**7**
arCOG00856	CaiC	I	Acyl-CoA synthetase (AMP-forming)/AMP-acid ligase II	**8**	**20**
arCOG01529	Acs	I	Acyl-coenzyme A synthetase/AMP-(fatty) acid ligase	**8**	**8**
arCOG02699	AccB	I	Biotin carboxyl carrier protein	**5**	**5**
arCOG01590	AccC	I	Biotin carboxylase	**6**	**15**
arCOG01879		I	Dolichol kinase family protein	**10**	**4**
arCOG00239	CaiD	I	Enoyl-CoA hydratase/carnithine racemase	**8**	**8**
arCOG03058	PgpB	I	Membrane-associated phospholipid phosphatase	**3**	**4**
arCOG04232	Sbm	I	Methylmalonyl-CoA mutase	**4**	**12**
arCOG01710	Sbm	I	Methylmalonyl-CoA mutase, C-terminal domain/subunit (cobalamin-binding)	**4**	**12**
arCOG01842	-	I	Putative sterol carrier protein	**6**	**5**
arCOG01260	FabG	I	Short-chain alcohol dehydrogenase	**5**	**4**
arCOG06042	TruB	J	DKCLD domain, TruB/PUA associated	**3**	**0**
arCOG01346	-	J	Predicted RNA-binding protein containing KH domain, possibly ribosomal protein	**7**	**22**
arCOG01630	TdcF	J	Putative translation initiation inhibitor, yjgF family	**11**	**10**
arCOG04057		J	Ribosomal L38E	**8**	**0**
arCOG04345	RPR2	J	RNase P subunit RPR2	**6**	**27**
arCOG01366	POP5	J	RNase P/RNase MRP subunit POP5	**4**	**0**
arCOG01124	GCD2	J	Translation initiation factor 2B subunit, eIF-2B alpha/beta/delta family	**3**	**19**

arCOG01125	GCD2	J	Translation initiation factor 2B subunit, eIF-2B alpha/beta/delta family	**7**	**5**
arCOG00974	Sun	J	tRNA or rRNA cytosine-C5-methylase	**3**	**6**
arCOG00826	WecD	K	Acetyltransferase (GNAT) family	**10**	**25**
arCOG00874	SSL2	K	DNA or RNA helicase of superfamily II	**11**	**16**
arCOG01361	ELP3	K	Histone acetyltransferase	**9**	**27**
arCOG02099	TroR	K	Mn-dependent transcriptional regulator (DtxR family)	**5**	**20**
arCOG04248	SIR2	K	NAD-dependent protein deacetylase, SIR2 family	**11**	**9**
arCOG01875		K	ParB-like nuclease domain	**7**	**4**
arCOG02217		K	Predicted antitoxin associated with PIN domain	**7**	**6**
arCOG03846	-	K	Predicted antitoxin, copG family	**5**	**1**
arCOG00374	-	K	Predicted membrane-associated trancriptional regulator	**9**	**4**
arCOG00393	-	K	Predicted membrane-associated trancriptional regulator	**9**	**0**
arCOG00743		K	Predicted transcriptional regulator	**2**	**4**
arCOG01055	-	K	Predicted transcriptional regulator	**9**	**11**
arCOG02242	-	K	Predicted transcriptional regulator	**5**	**13**
arCOG03358	-	K	Predicted transcriptional regulator	**5**	**0**
arCOG03879	-	K	Predicted transcriptional regulator	**4**	**0**
arCOG05900	-	K	Predicted transcriptional regulator	**6**	**0**
arCOG07512	-	K	Predicted transcriptional regulator	**2**	**0**
arCOG00610	-	K	Predicted transcriptional regulator, contains C-terminal CBS domains	**11**	**20**
arCOG02271	-	K	Predicted transcriptional regulator, c-terminal HTH-like domain	**7**	**6**
arCOG02272	-	K	Predicted transcriptional regulator, c-terminal HTH-like domain	**6**	**8**
arCOG00001	-	K	Predicted transcriptional regulator, PadR family	**10**	**14**
arCOG00999	LSM1	K	Small nuclear ribonucleoprotein (snRNP) homolog	**10**	**0**
arCOG02038	-	K	Sugar-specific transcriptional regulator TrmB	**5**	**11**
arCOG01128	TenA	K	Transcriptional activator TenA	**10**	**9**
arCOG01585	Lrp	K	Transcriptional regulator (Lrp/AsnC family)	**6**	**13**
arCOG00812	AbrB	K	Transcriptional regulator AbrB	**3**	**3**
arCOG00815	AbrB	K	Transcriptional regulator AbrB	**6**	**6**
arCOG00818	AbrB	K	Transcriptional regulator AbrB	**3**	**5**
arCOG00820	AbrB	K	Transcriptional regulator AbrB	**6**	**0**
arCOG00731	ArsR	K	Transcriptional regulator containing HTH domain, ArsR family	**7**	**6**
arCOG01679	ArsR	K	Transcriptional regulator containing HTH domain, ArsR family	**7**	**8**
arCOG03859	ArsR	K	Transcriptional regulator containing HTH domain, ArsR family	**7**	**0**
arCOG04056	ArsR	K	Transcriptional regulator containing HTH domain, ArsR family	**7**	**0**
arCOG05989		K	Transcriptional regulator, xre family	**5**	**0**

arCOG00889	-	L	Adenine-specific DNA methylase containing a Zn-ribbon	**4**	**7**
arCOG04200		L	CC1 family DNA binding protein	**6**	**0**
arCOG00329	PolB	L	DNA polymerase elongation subunit (family B)	**4**	**7**
arCOG00282	-	L	HerA helicase	**7**	**0**
arCOG00285	-	L	HerA helicase	**10**	**0**
arCOG04155		L	HhH-GPD superfamily base excision DNA repair protein	**8**	**0**
arCOG01072	MutT	L	NUDIX family hydrolase	**9**	**8**
arCOG01073	MutT	L	NUDIX family hydrolase	**11**	**7**
arCOG01083	MutT	L	NUDIX family hydrolase	**2**	**5**
arCOG01442	-	L	Predicted HD superfamily hydrolase, possibly a nuclease	**5**	**2**
arCOG01304	-	L	Predicted nuclease of the RecB family	**11**	**15**
arCOG03164	-	L	Predicted site-specific integrase-resolvase	**6**	**8**
arCOG00417	RecA	L	RecA/RadA recombinase	**3**	**26**
arCOG04294	RecA	L	RecA/RadA recombinase	**5**	**0**
arCOG01247	XerD	L	Site-specific recombinase XerD	**3**	**1**
arCOG00679	-	L	Transposase	**9**	**13**
arCOG05935	-	L	Type IV secretory pathway, VirB4 component	**6**	**0**
arCOG00465	-	L	Uncharacterized protein related to Endonuclease III	**7**	**0**
arCOG04188	RfbC	M	dTDP-4-dehydrorhamnose 3,5-epimerase or related enzyme	**7**	**17**
arCOG01367	RfbD	M	dTDP-4-dehydrorhamnose reductase	**7**	**17**
arCOG01371	RfbB	M	dTDP-D-glucose 4,6-dehydratase	**5**	**16**
arCOG00895	WcaA	M	Glycosyltransferase	**4**	**22**
arCOG01381	WcaA	M	Glycosyltransferase	**10**	**20**
arCOG01389	-	M	Glycosyltransferase	**10**	**20**
arCOG01407	RfaG	M	Glycosyltransferase	**8**	**14**
arCOG01408	RfaG	M	Glycosyltransferase	**5**	**6**
arCOG01417	RfaG	M	Glycosyltransferase	**7**	**2**
arCOG00609	-	M	Predicted membrane-associated Zn-dependent protease	**2**	**18**
arCOG00059	AgaS	M	Predicted phosphosugar isomerase	**4**	**0**
arCOG00673	-	M	Predicted sugar nucleotidyltransferase	**3**	**8**
arCOG02091	-	M	S-layer domain	**2**	**0**
arCOG01809	FlaJ	N	Archaeal flagella assembly protein J	**4**	**20**
arCOG01829	FlaB	N	Archaeal flagellins	**4**	**20**
arCOG00453	-	N	Conserved protein implicated in secretion	**6**	**0**
arCOG01812	TadC	N	Flp pilus assembly protein TadC	**8**	**13**
arCOG04148	FlaH	N	Predicted ATPase involved in biogenesis of archaeal flagella	**4**	**20**

arCOG01824	FlaF	N	Putative archaeal flagellar protein F	**4**	**20**
arCOG01822	FlaG	N	Putative archaeal flagellar protein G	**3**	**20**
arCOG00299		O	DsbA family protein	**4**	**0**
arCOG02007	-	O	Highly conserved protein containing a thioredoxin domain	**6**	**17**
arCOG01915	HflC	O	Membrane protease subunit, stomatin/prohibitin homolog	**12**	**26**
arCOG01912	-	O	Membrane protein implicated in regulation of membrane protease activity	**5**	**16**
arCOG01832	IbpA	O	Molecular chaperone (HSP20 family)	**10**	**27**
arCOG00310	Bcp	O	Peroxiredoxin	**10**	**17**
arCOG00479	CyoE	O	Polyprenyltransferase (cytochrome oxidase assembly factor)	**11**	**8**
arCOG03686	-	O	Predicted redox protein, regulator of disulfide bond formation	**5**	**8**
arCOG04235	-	O	Predicted thioredoxin/glutaredoxin	**12**	**0**
arCOG05850	Pcp	O	Pyrrolidone-carboxylate peptidase (N-terminal pyroglutamyl peptidase)	**5**	**4**
arCOG00936	PflA	O	Pyruvate-formate lyase-activating enzyme	**6**	**4**
arCOG00947	PflA	O	Pyruvate-formate lyase-activating enzyme	**6**	**1**
arCOG00065	csdA	O	Selenocysteine lyase/Cysteine desulfurase	**7**	**19**
arCOG01910	NfeD	O	Short-chain alcohol dehydrogenase	**9**	**13**
arCOG00704	AprE	O	Ssurface layer-associated subtilisin-like serine protease	**7**	**0**
arCOG01975	TrxA	O	Thiol-disulfide isomerase or thioredoxin	**2**	**4**
arCOG06075	TrxA	O	Thiol-disulfide isomerase or thioredoxin	**4**	**0**
arCOG07518	TrxA	O	Thiol-disulfide isomerase or thioredoxin	**2**	**0**
arCOG05634	FdhE	O	Uncharacterized protein involved in formate dehydrogenase formation	**3**	**0**
arCOG01929	XdhC	O	Xanthine and CO dehydrogenase maturation factor, XdhC/CoxF family	**8**	**4**
arCOG02248	CbiM	P	ABC-type Co2+ transport system, permease component	**6**	**21**
arCOG03159	CbiM	P	ABC-type Co2+ transport system, permease component	**6**	**2**
arCOG00188	CbiO	P	ABC-type cobalt transport system, ATPase component	**9**	**11**
arCOG00202	CbiO	P	ABC-type cobalt transport system, ATPase component	**6**	**24**
arCOG02252	CbiQ	P	ABC-type cobalt transport system, permease component CbiQ or related transporter	**3**	**0**
arCOG00221	AfuA	P	ABC-type Fe3+ transport system, periplasmic component	**7**	**1**
arCOG00163	ThiP	P	ABC-type Fe3+ transport system, permease component	**7**	**10**
arCOG05372	ZnuC	P	ABC-type Mn/Zn transport system, ATPase component	**6**	**2**
arCOG00193	TauB	P	ABC-type nitrate/sulfonate/bicarbonate transport system, ATPase component	**10**	**19**
arCOG01803	TauA	P	ABC-type nitrate/sulfonate/bicarbonate transport system, periplasmic component	**3**	**16**
arCOG00169	TauC	P	ABC-type nitrate/sulfonate/bicarbonate transport system, permease component	**3**	**16**
arCOG01576	ZntA	P	Cation transport ATPase	**11**	**24**
arCOG01868	Fur	P	Fe2+/Zn2+ uptake regulation protein, fur/PerR	**10**	**7**
arCOG01953	KefB	P	Kef-type K+ transport system, membrane component	**7**	**20**

arCOG02850	ArsA	P	Oxyanion-translocating ATPase	**2**	**0**
arCOG00230	-	P	Periplasmic molybdate-binding protein/domain	**7**	**1**
arCOG02640	-	P	Phosphate transport regulator (distant homolog of PhoU)	**4**	**14**
arCOG02267	PitA	P	Phosphate/sulphate permease	**6**	**22**
arCOG01474	MMT1	P	Predicted Co/Zn/Cd cation transporter	**9**	**22**
arCOG01479	MMT1	P	Predicted Co/Zn/Cd cation transporter	**2**	**0**
arCOG00576	-	P	Predicted divalent heavy-metal cations transporter	**7**	**1**
arCOG00577	-	P	Predicted divalent heavy-metal cations transporter	**5**	**17**
arCOG02019	SseA	P	Rhodanese-related sulfurtransferase	**7**	**7**
arCOG04147	SodA	P	Superoxide dismutase	**9**	**14**
arCOG00235	MhpD	Q	2-keto-4-pentenoate hydratase/2-oxohepta-3-ene-1,7-dioic acid hydratase (catechol pathway)	**10**	**24**
arCOG02004	-	Q	Maleate cis-trans isomerase	**4**	**5**
arCOG01521	-	Q	Phytoene dehydrogenase or related enzyme	**5**	**18**
arCOG01400	SmtA	Q	SAM-dependent methyltransferase	**8**	**5**
arCOG03773	SmtA	Q	SAM-dependent methyltransferase	**8**	**0**
arCOG07511	SmtA	Q	SAM-dependent methyltransferase, fragment?	**2**	**0**
arCOG02441	NosY	R	ABC-type transport system involved in multi-copper enzyme maturation, permease component	**4**	**0**
arCOG00186	-	R	ABC-type uncharacterized transport system, ATPase component	**8**	**8**
arCOG00259	-	R	ABC-type uncharacterized transport system, permease component	**6**	**0**
arCOG00260	-	R	ABC-type uncharacterized transport system, permease component	**7**	**8**
arCOG01618	ARA1	R	Aldo/keto reductase, related to diketogulonate reductase	**9**	**0**
arCOG01648	MhpC	R	Alpha/beta superfamily hydrolase	**10**	**16**
arCOG00662	-	R	Biotin synthase-related enzyme	**5**	**13**
arCOG03031	ywfI	R	Chlorite dismutase	**4**	**7**
arCOG04210	-	R	Enzyme related to GTP cyclohydrolase I	**6**	**0**
arCOG02291	-	R	HAD superfamily hydrolase	**11**	**23**
arCOG04230	-	R	HD supefamily hydrolase	**7**	**8**
arCOG03721		R	HEPN domain	**10**	**0**
arCOG03722		R	HEPN domain	**9**	**0**
arCOG06083		R	Imidazolonepropionase family metallohydrolase	**2**	**0**
arCOG00754	-	R	Malate/quinone oxidoreductase or related dehydrogenase	**3**	**5**
arCOG03682	SPS1	R	Membrane associated serine/threonine protein kinase	**9**	**2**
arCOG06097		R	Membrane protein, containing cytochrome c-like domain	**2**	**0**
arCOG00500	ElaC	R	Metal-dependent hydrolase of the beta-lactamase superfamily	**11**	**13**
arCOG00499	PhnP	R	Metal-dependent hydrolase of the beta-lactamase superfamily	**0**	**24**
arCOG00441	-	R	MoxR-like ATPase	**7**	**9**

arCOG04466	-	R	Na+-dependent transporter of the SNF family	**2**	**14**
arCOG01299	HcaD	R	NAD(FAD)-dependent dehydrogenase	**4**	**4**
arCOG04215	-	R	NH2-acetyltransferase	**3**	**3**
arCOG01995		R	Oligoketide cyclase/lipid transport protein family	**6**	**3**
arCOG02293	-	R	Phosphatase/phosphohexomutase HAD superfamily	**9**	**9**
arCOG04702	-	R	Phospholipid-binding protein	**8**	**19**
arCOG02214	RfbX	R	Polysaccharide biosynthesis protein, Mvin family	**6**	**0**
arCOG03647	-	R	Predicted acetyltransferase	**3**	**5**
arCOG06090	-	R	Predicted acetyltransferase	**2**	**0**
arCOG02303	SurE	R	Predicted acid phosphatase	**8**	**22**
arCOG02959	Iap	R	Predicted aminopeptidase	**3**	**9**
arCOG00887	-	R	Predicted ATPase (AAA+ superfamily)	**4**	**4**
arCOG04052	-	R	Predicted cation transporter	**5**	**4**
arCOG04227	-	R	Predicted CoA-binding protein	**7**	**14**
arCOG02238	-	R	Predicted DNA-binding protein	**4**	**18**
arCOG04212	-	R	Predicted DNA-binding protein with PD1-like DNA-binding motif	**4**	**21**
arCOG01229	-	R	Predicted GTPase	**8**	**5**
arCOG01860	-	R	Predicted HD superfamily hydrolase	**3**	**16**
arCOG01215	-	R	Predicted hydrolase (HAD superfamily)	**2**	**4**
arCOG01350	-	R	Predicted inorganic polyphosphate/ATP-NAD kinase	**6**	**14**
arCOG05320		R	Predicted membrane-bound metal-dependent hydrolase	**6**	**1**
arCOG03705	-	R	Predicted metal-dependent hydrolase	**9**	**3**
arCOG01930	-	R	Predicted metal-dependent hydrolase of the TIM-barrel fold	**4**	**1**
arCOG01931	-	R	Predicted metal-dependent hydrolase of the TIM-barrel fold	**5**	**7**
arCOG01254	-	R	Predicted metal-dependent hydrolase related to alanyl-tRNA synthetase HxxxH domain	**11**	**13**
arCOG01138	-	R	Predicted metal-dependent protease of the PAD1/JAB1 superfamily	**7**	**8**
arCOG04217	-	R	Predicted metallopeptidase	**11**	**0**
arCOG01283	-	R	Predicted nucleic-acid-binding protein containing a Zn-ribbon	**8**	**3**
arCOG01287	-	R	Predicted nucleic-acid-binding protein containing a Zn-ribbon	**6**	**4**
arCOG01288	-	R	Predicted nucleic-acid-binding protein containing a Zn-ribbon	**6**	**1**
arCOG01204	-	R	Predicted nucleotidyltransferase	**9**	**3**
arCOG01205	-	R	Predicted nucleotidyltransferase	**11**	**6**
arCOG04046	-	R	Predicted phosphatase	**6**	**2**
arCOG01144	Icc	R	Predicted phosphohydrolase	**7**	**2**
arCOG04328	-	R	Predicted P-loop ATPase/GTPase	**6**	**5**
arCOG04331	-	R	Predicted thioesterase	**6**	**1**

arCOG01093	-	R	Protein distantly related to bacterial ferritins	**5**	**8**
arCOG04082	-	R	Protein related to penicillin acylase	**5**	**6**
arCOG05825	-	R	Radical SAM superfamily enzyme	**5**	**4**
arCOG02284	-	R	Secreted protein containing C-terminal beta-propeller domain distantly related to WD-40 repeats	**5**	**7**
arCOG00264	-	R	Sulfite oxidase or related enzyme	**11**	**7**
arCOG01137	FcbC	R	Thioesterase superfamily enzyme	**8**	**13**
arCOG03038	NrfG	R	TPR repeats containing protein	**4**	**12**
arCOG04197		R	Transposase related protein	**4**	**1**
arCOG00257	Med	R	Uncharacterized ABC-type transport system, periplasmic component/surface lipoprotein	**6**	**2**
arCOG00258	Med	R	Uncharacterized ABC-type transport system, periplasmic component/surface lipoprotein	**7**	**7**
arCOG00261	-	R	Uncharacterized ABC-type transport system, permease component	**7**	**8**
arCOG00262	-	R	Uncharacterized ABC-type transport system, permease component	**5**	**0**
arCOG00932	-	R	Uncharacterized conserved protein related to pyruvate formate-lyase activating enzyme	**10**	**22**
arCOG02742	-	R	Uncharacterized conserved protein, DUF58 family, contains vWF domain	**5**	**11**
arCOG04284	yhhQ	R	Uncharacterized member of the PurR regulon	**7**	**9**
arCOG01873	-	R	Uncharacterized MobA-related protein	**9**	**6**
arCOG00442	-	R	Uncharacterized protein containing a von Willebrand factor type A (vWA) domain	**11**	**4**
arCOG02900	-	R	Uncharacterized protein containing a von Willebrand factor type A (vWA) domain	**5**	**5**
arCOG04285	PrpD	R	Uncharacterized protein involved in propionate catabolism	**9**	**5**
arCOG01455	AdhP	R	Zn-dependent alcohol dehydrogenase	**11**	**9**
arCOG00498	GloB	R	Zn-dependent hydrolase, glyoxylase family	**10**	**10**
arCOG05978		S	Extracellular protein containing Kelch and FN3 domains	**4**	**3**
arCOG01191	-	S	HEPN domain containing protein	**11**	**10**
arCOG01615	-	S	Predicted membrane protein	**10**	**7**
arCOG02008	-	S	Predicted membrane protein	**12**	**10**
arCOG02159	-	S	Predicted membrane protein	**9**	**12**
arCOG02228	-	S	Predicted membrane protein	**4**	**10**
arCOG02718	-	S	Predicted membrane protein	**7**	**5**
arCOG03749	-	S	Predicted membrane protein	**9**	**1**
arCOG04203	-	S	Predicted membrane protein	**2**	**12**
arCOG00287		S	Predicted NurA-like nuclease	**10**	**0**
arCOG03765		S	Uncharacterized conserved membrane protein	**8**	**0**
arCOG07272		S	Uncharacterized conserved membrane protein	**5**	**2**
arCOG01572		S	Uncharacterized conserved membrane protein	**9**	**5**
arCOG00022		S	Uncharacterized conserved protein	**3**	**1**
arCOG00372		S	Uncharacterized conserved protein	**3**	**5**

arCOG00425	S	Uncharacterized conserved protein	**3**	**0**
arCOG01310	S	Uncharacterized conserved protein	**6**	**1**
arCOG01315	S	Uncharacterized conserved protein	**2**	**0**
arCOG01806	S	Uncharacterized conserved protein	**3**	**4**
arCOG02103	S	Uncharacterized conserved protein	**10**	**4**
arCOG02218	S	Uncharacterized conserved protein	**7**	**0**
arCOG03129	S	Uncharacterized conserved protein	**3**	**3**
arCOG03754	S	Uncharacterized conserved protein	**5**	**3**
arCOG03820	S	Uncharacterized conserved protein	**2**	**4**
arCOG03824	S	Uncharacterized conserved protein	**3**	**3**
arCOG03838	S	Uncharacterized conserved protein	**4**	**4**
arCOG03872	S	Uncharacterized conserved protein	**3**	**0**
arCOG03882	S	Uncharacterized conserved protein	**6**	**0**
arCOG04024	S	Uncharacterized conserved protein	**2**	**2**
arCOG04025	S	Uncharacterized conserved protein	**5**	**2**
arCOG04033	S	Uncharacterized conserved protein	**3**	**1**
arCOG04039	S	Uncharacterized conserved protein	**6**	**1**
arCOG04079	S	Uncharacterized conserved protein	**2**	**4**
arCOG04104	S	Uncharacterized conserved protein	**4**	**0**
arCOG04141	S	Uncharacterized conserved protein	**3**	**0**
arCOG04158	S	Uncharacterized conserved protein	**2**	**3**
arCOG04165	S	Uncharacterized conserved protein	**4**	**0**
arCOG04211	S	Uncharacterized conserved protein	**3**	**0**
arCOG04216	S	Uncharacterized conserved protein	**4**	**0**
arCOG04259	S	Uncharacterized conserved protein	**3**	**1**
arCOG04267	S	Uncharacterized conserved protein	**4**	**0**
arCOG04268	S	Uncharacterized conserved protein	**5**	**0**
arCOG04275	S	Uncharacterized conserved protein	**4**	**0**
arCOG04283	S	Uncharacterized conserved protein	**7**	**0**
arCOG04326	S	Uncharacterized conserved protein	**3**	**0**
arCOG05371	S	Uncharacterized conserved protein	**5**	**2**
arCOG05401	S	Uncharacterized conserved protein	**6**	**3**
arCOG05472	S	Uncharacterized conserved protein	**6**	**0**
arCOG05561	S	Uncharacterized conserved protein	**7**	**0**
arCOG05906	S	Uncharacterized conserved protein	**4**	**0**
arCOG05924	S	Uncharacterized conserved protein	**4**	**0**

arCOG05988		S	Uncharacterized conserved protein	**4**	**0**
arCOG06022		S	Uncharacterized conserved protein	**7**	**0**
arCOG06071		S	Uncharacterized conserved protein	**4**	**0**
arCOG06076		S	Uncharacterized conserved protein	**2**	**0**
arCOG06081		S	Uncharacterized conserved protein	**2**	**0**
arCOG06082		S	Uncharacterized conserved protein	**2**	**0**
arCOG06087		S	Uncharacterized conserved protein	**2**	**0**
arCOG06088		S	Uncharacterized conserved protein	**4**	**0**
arCOG06092		S	Uncharacterized conserved protein	**2**	**0**
arCOG06094		S	Uncharacterized conserved protein	**2**	**0**
arCOG06095		S	Uncharacterized conserved protein	**2**	**0**
arCOG06096		S	Uncharacterized conserved protein	**4**	**0**
arCOG06098		S	Uncharacterized conserved protein	**2**	**0**
arCOG06100		S	Uncharacterized conserved protein	**3**	**0**
arCOG06101		S	Uncharacterized conserved protein	**3**	**0**
arCOG06102		S	Uncharacterized conserved protein	**3**	**0**
arCOG06104		S	Uncharacterized conserved protein	**2**	**0**
arCOG06105		S	Uncharacterized conserved protein	**2**	**0**
arCOG06689		S	Uncharacterized conserved protein	**2**	**1**
arCOG07192		S	Uncharacterized conserved protein	**4**	**0**
arCOG07300		S	Uncharacterized conserved protein	**3**	**0**
arCOG07331		S	Uncharacterized conserved protein	**4**	**0**
arCOG07459		S	Uncharacterized conserved protein	**2**	**0**
arCOG07462		S	Uncharacterized conserved protein	**2**	**0**
arCOG07463		S	Uncharacterized conserved protein	**2**	**0**
arCOG07464		S	Uncharacterized conserved protein	**2**	**0**
arCOG07493		S	Uncharacterized conserved protein	**3**	**0**
arCOG07515		S	Uncharacterized conserved protein	**2**	**0**
arCOG07516		S	Uncharacterized conserved protein	**2**	**0**
arCOG07753		S	Uncharacterized conserved protein	**2**	**0**
arCOG07754		S	Uncharacterized conserved protein	**2**	**0**
arCOG07755		S	Uncharacterized conserved protein	**2**	**0**
arCOG07864		S	Uncharacterized conserved protein	**2**	**0**
arCOG07865		S	Uncharacterized conserved protein	**2**	**0**
arCOG00021	-	S	Uncharacterized conserved protein	**11**	**13**
arCOG00723	-	S	Uncharacterized conserved protein	**8**	**4**

arCOG00761	-	S	Uncharacterized conserved protein	**11**	**19**
arCOG01699	-	S	Uncharacterized conserved protein	**10**	**0**
arCOG01713	-	S	Uncharacterized conserved protein	**6**	**2**
arCOG02118	-	S	Uncharacterized conserved protein	**2**	**0**
arCOG02287	-	S	Uncharacterized conserved protein	**6**	**18**
arCOG03217	-	S	Uncharacterized conserved protein	**4**	**0**
arCOG03229	-	S	Uncharacterized conserved protein	**5**	**13**
arCOG04058	-	S	Uncharacterized conserved protein	**3**	**18**
arCOG04298	-	S	Uncharacterized conserved protein	**12**	**10**
arCOG04507	-	S	Uncharacterized conserved protein	**9**	**8**
arCOG04733	-	S	Uncharacterized conserved protein	**5**	**22**
arCOG07519		S	Uncharacterized conserved protein	**2**	**0**
arCOG01422	-	S	Uncharacterized conserved protein containing a coiled-coil domain	**9**	**0**
arCOG04374	-	S	Uncharacterized conserved protein related to dihydrodipicolinate reductase	**3**	**1**
arCOG01994	SpoIIM	S	Uncharacterized membrane protein	**3**	**22**
arCOG00317	-	S	Uncharacterized protein conserved in archaea	**8**	**3**
arCOG01224	-	S	Uncharacterized protein conserved in archaea	**12**	**27**
arCOG01244	-	S	Uncharacterized protein conserved in archaea	**5**	**2**
arCOG01302	-	S	Uncharacterized protein conserved in archaea	**12**	**14**
arCOG01668	-	S	Uncharacterized protein conserved in archaea	**7**	**5**
arCOG03772	-	S	Uncharacterized protein conserved in archaea	**10**	**4**
arCOG03880	-	S	Uncharacterized protein conserved in archaea	**2**	**4**
arCOG04123	-	S	Uncharacterized protein conserved in archaea	**12**	**0**
arCOG04132	-	S	Uncharacterized protein conserved in archaea	**5**	**4**
arCOG04085	-	S	Uncharacterized protein conserved in bacteria	**5**	**0**
arCOG03460	-	S	Uncharacterized protein, LmbE homolog	**5**	**9**
arCOG06080		S	vWFA domain containing protein	**3**	**0**
arCOG05332	-	T	FHA domain containing protein	**9**	**1**
arCOG00449	UspA	T	Nucleotide-binding protein, UspA family	**3**	**18**
arCOG03413	CDC14	T	Predicted protein-tyrosine phosphatase	**10**	**10**
arCOG01174	RAD55	T	RecA-superfamily ATPase implicated in signal transduction	**7**	**10**
arCOG01148	ApaH	T	Serine/threonine protein phosphatase PP2A family	**3**	**17**
arCOG01463	-	V	ABC-type multidrug transport system, permease component	**5**	**14**
arCOG01465	-	V	ABC-type multidrug transport system, permease component	**10**	**8**
arCOG00771	AmpC	V	Beta-lactamase class C and other penicillin binding protein	**4**	**7**
arCOG02658	-	V	CRISPR system related protein, RAMP superfamily	**8**	**8**

arCOG03779	McrB	V	GTPase subunit of restriction endonuclease	**9**	**0**
arCOG01731	NorM	V	Na+-driven multidrug efflux pump	**3**	**20**
arCOG00713	-	V	PIN domain containing protein	**5**	**4**
arCOG00726	-	V	PIN domain containing protein	**8**	**3**
arCOG00727	-	V	PIN domain containing protein	**7**	**0**
arCOG00729	-	V	PIN domain containing protein	**8**	**0**
arCOG02730	-	V	PIN domain containing protein	**6**	**5**
arCOG02731	VapC	V	PIN domain containing protein	**3**	**0**
arCOG06028	VapC	V	PIN domain containing protein	**6**	**0**
arCOG02219	VapC	V	Predicted nucleic acid-binding protein, contains PIN domain	**6**	**14**

Table S3. Functional gene categories (arCOGs) present in *N. equitans* but absent in *I. hospitalis* and their distribution in archaeal genomes

**arCOG**	**Gene**	**Type**	**Annotation**	**CREN**	**EURY**	**Nano protein ID**
arCOG02200	Hmp	C	Flavodoxin reductase (ferredoxin-NADPH reductase) family 1	**0**	**7**	**NEQ051**
arCOG02201	FtsZ	D	Cell division GTPase	**0**	**26**	**NEQ133**
arCOG02297	IlvE	E	Branched-chain amino acid aminotransferase/4-amino-4-deoxychorismate lyase	**3**	**20**	**NEQ190**
arCOG01352	GdhA	E	Glutamate dehydrogenase/leucine dehydrogenase	**12**	**19**	**NEQ077**
arCOG04247	-	E	Zn-dependent carboxypeptidase	**11**	**9**	**NEQ042**
arCOG00438		R	AAA family ATPase	**1**	**17**	**NEQ401**
arCOG00546	-	J	mRNA degradation ribonuclease J1/J2 (metallo-beta-lactamase superfamily)	**0**	**23**	**NEQ466**
arCOG01346	-	J	Predicted RNA-binding protein containing KH domain, possibly ribosomal protein	**7**	**22**	**NEQ428**

arCOG04449	TruA	J	Pseudouridylate synthase	**4**	**27**	**NEQ333**
arCOG00990	-	J	Queuine tRNA-ribosyltransferase, contain PUA domain	**1**	**26**	**NEQ526**
arCOG00122	TrmA	J	SAM-dependent methyltransferase related to tRNA (uracil-5-)-methyltransferase	**0**	**4**	**NEQ053**
arCOG02037	-	K	Sugar-specific transcriptional regulator TrmB	**8**	**21**	**NEQ098**
arCOG04447	-	L	Archaeal DNA polymerase II, large subunit	**0**	**27**	**NEQ420**
arCOG04455	HYS2	L	Archaeal DNA polymerase II, small subunit/DNA polymerase delta, subunit B	**0**	**26**	**NEQ240**
arCOG00331	PolB	L	DNA polymerase elongation subunit (family B)	**0**	**2**	**NEQ528**
arCOG00872	MPH1	L	ERCC4-like helicase	**0**	**26**	**NEQ387**
arCOG02144	HHT1	L	Histones H3 and H4	**2**	**24**	**NEQ288**
arCOG00424	RecJ	L	Single-stranded DNA-specific exonuclease RecJ	**5**	**5**	**NEQ118**
arCOG00787	UvrD	L	Superfamily I DNA and RNA helicase	**0**	**3**	**NEQ549**
arCOG01381	WcaA	M	Glycosyltransferase	**10**	**20**	**NEQ172**
arCOG01812	TadC	N	Flp pilus assembly protein TadC	**8**	**13**	**NEQ268**
arCOG01818	VirB11	N	Type IV secretory pathway, VirB11 component, or related ATPase involved in archaeal flagella biosynthesis	**4**	**13**	**NEQ425**
arCOG01716	sufB	O	Cysteine desulfurase activator SufB	**0**	**6**	**NEQ129**
arCOG01832	IbpA	O	Molecular chaperone (HSP20 family)	**10**	**27**	**NEQ344**
arCOG01311	SppA	O	Periplasmic serine protease (ClpP class)	**5**	**20**	**NEQ315**
arCOG02160	LonB	O	Predicted ATP-dependent protease	**0**	**26**	**NEQ349**
arCOG01188	-	O	Predicted carbamoyl transferase, NodU family	**1**	**8**	**NEQ127**
arCOG02881	ECM27	P	Ca2+/Na+ antiporter	**8**	**23**	**NEQ486**
arCOG02265	CorA	P	Mg2+ and Co2+ transporter	**2**	**16**	**NEQ501**
arCOG04355	Mae	C	mae1-like C4-dicarboxylate transporter	**1**	**7**	**NEQ014**
arCOG04347	SmtA	Q	SAM-dependent methyltransferase	**1**	**13**	**NEQ490**
arCOG00601	-	R	CBS domain	**5**	**18**	**NEQ084**
arCOG04702	-	R	Phospholipid-binding protein	**8**	**19**	**NEQ159**
arCOG02209	RfbX	R	Polysaccharide biosynthesis protein, Mvin family	**4**	**27**	**NEQ090**
arCOG01204	-	R	Predicted nucleotidyltransferase	**9**	**3**	**NEQ050**
arCOG02642	yhhT	R	Predicted permease, member of the PurR regulon	**0**	**22**	**NEQ162**
arCOG02292	Gph	R	Predicted phosphatase	**1**	**18**	**NEQ340**
arCOG03450		R	Predicted surface protease of transglutaminase family	**0**	**8**	**NEQ353**
arCOG04065	PqqL	R	Predicted Zn-dependent peptidase	**6**	**0**	**NEQ023**
arCOG02155	-	R	Protein implicated in RNA metabolism, contains PRC-barrel domain	**0**	**26**	**NEQ258**
arCOG02742	-	R	Uncharacterized conserved protein, DUF58 family, contains vWF domain	**5**	**11**	**NEQ232**
arCOG02900	-	R	Uncharacterized protein containing a von Willebrand factor type A (vWA) domain	**5**	**5**	**NEQ403**
arCOG01378	-	R	Uncharacterized protein of the AP superfamily	**5**	**9**	**NEQ128**

arCOG02737	-	S	NifX family protein	**4**	**3**	**NEQ386**
arCOG04469	-	S	Predicted membrane protein	**0**	**24**	**NEQ455**
arCOG02177	-	S	Predicted membrane protein	**0**	**22**	**NEQ352**
arCOG06945		S	Uncharacterized conserved protein	**1**	**0**	**NEQ026**
arCOG06946		S	Uncharacterized conserved membrane protein	**1**	**0**	**NEQ415**
arCOG01814		S	Uncharacterized conserved membrane protein	**0**	**8**	**NEQ285**
arCOG00428		S	Protein similar to exonuclease RecJ central domain	**0**	**4**	**NEQ104**
arCOG05748		S	Uncharacterized conserved protein	**0**	**4**	**NEQ298**
arCOG02263	-	S	Uncharacterized conserved protein	**0**	**24**	**NEQ277**
arCOG02285	-	S	Uncharacterized conserved protein	**0**	**5**	**NEQ310**
arCOG03055	SecD	U	Preprotein translocase subunit SecD	**0**	**23**	**NEQ436**
arCOG03054	SecF	U	Preprotein translocase subunit SecF	**0**	**23**	**NEQ437**
arCOG03617	-	V	CRISPR system related protein	**1**	**6**	**NEQ019**
arCOG04342	-	V	CRISPR system related protein, RAMP superfamily	**0**	**14**	**NEQ001**
arCOG01445	-	V	CRISPR-associated helicase Cas3	**0**	**16**	**NEQ022**
arCOG00794	-	V	CRISPR-associated protein Cas4 (RecB family exonuclease)	**0**	**12**	**NEQ021**

Table S4. Gene family expansions in the *I. hospitalis* genome

WD-40 repeats	Igni_0008, Igni_0114, Igni_0419, Igni_0434, Igni_0435, Igni_0477, Igni_0541, Igni_0561, Igni_0808, Igni_1434
V4R	Igni_0701, Igni_0581, Igni_1239, Igni_1243, Igni_1332, Igni_0267, Igni_0597, Igni_0524, Igni_0644, (and maybe Igni_0643)
CBS domain containing proteins	Igni_1375, Igni_0444, Igni_0497, Igni_0648, Igni_0831, Igni_1319, Igni_0598, Igni_0655, Igni_1094, Igni_1212, Igni_1414, Igni_1222
Zn-ribbons	Igni_1432, Igni_0048, Igni_0593, Igni_0597
Cytochrome c	Igni_0759, Igni_1052
Uncharacterized	Igni_0370, Igni_0806
Thymidylate synthase, flavin-dependent	Igni_0023, Igni_1422
Uncharacterized	Igni_0267, Igni_1243
Membrane	Igni_0335, Igni_0336
Uncharacterized	Igni_0389, Igni_0727
Membrane	Igni_0520, Igni_1195
Fe-S?	Igni_0524, Igni_1243, Igni_0267
