# Supplementary material for: A genomic analysis of the archaeal system Ignicoccus hospitalis-Nanoarchaeum equitans
Source: Genome Biol. 2008 Nov 10;9(11):R158. doi: 10.1186/gb-2008-9-11-r158 (PMC2614490; doi:10.1186/gb-2008-9-11-r158)
Supplement: Additional data file 2 — Figure S1: a phylogenetic tree of cultivated thermophilic species of Crenarchaeota based on SSU rRNA sequences. Figure S2: phylogenetic trees of archaeal tyrosyl-tRNA synthetases and of family IV endonucleases. Figure S3: a phylogenetic tree of the alpha subunit of archaeal 2-oxoacid: ferredoxin oxidoreductases. Figure S4: amino acid-based sequence alignment of conserved regions of the alpha and beta subunits of pyruvate:ferredoxin oxidoreductases and OGORs. [file gb-2008-9-11-r158-S2.pdf]

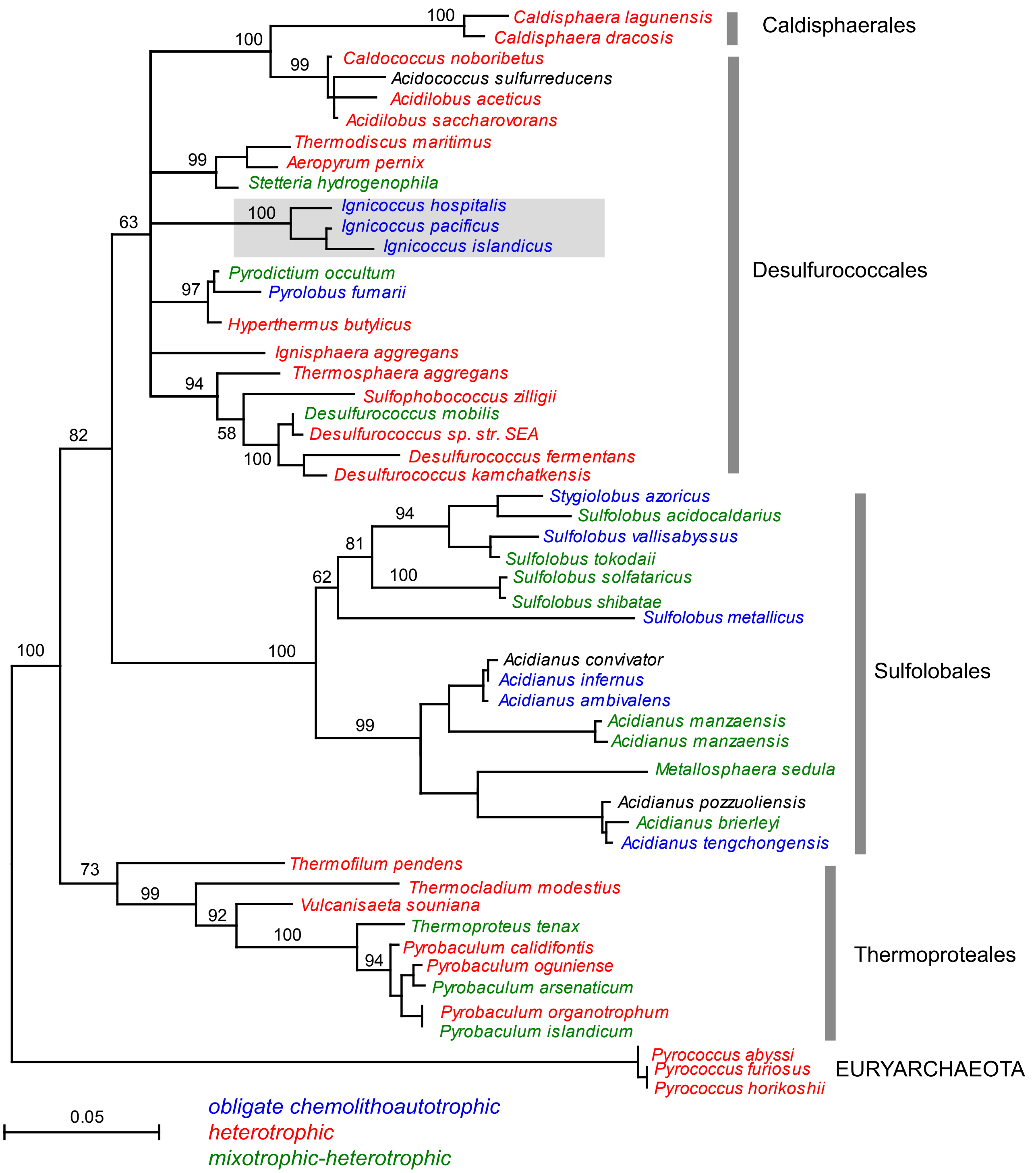

Figure S1. Maximum likelihood phylogenetic tree of cultivated thermophilic species of Crenarchaeota based on SSU rRNA sequences.The type of metabolism is indicated for each species based on oirginal species description literature [summarized in Stackebrandt E, Huber H, Schumann P, Stetter KO, Goodfellow M, et al. (2006) The Prokaryotes. New York: Springer]. Species for which no metabolic information was available are in black. The numbers indicate bootstrap support values, based on 100 replicates. Where the value was <50 the branches were collapsed. The shaded area indicates the place of *Ignicoccus* within the order Desulfurococcales.

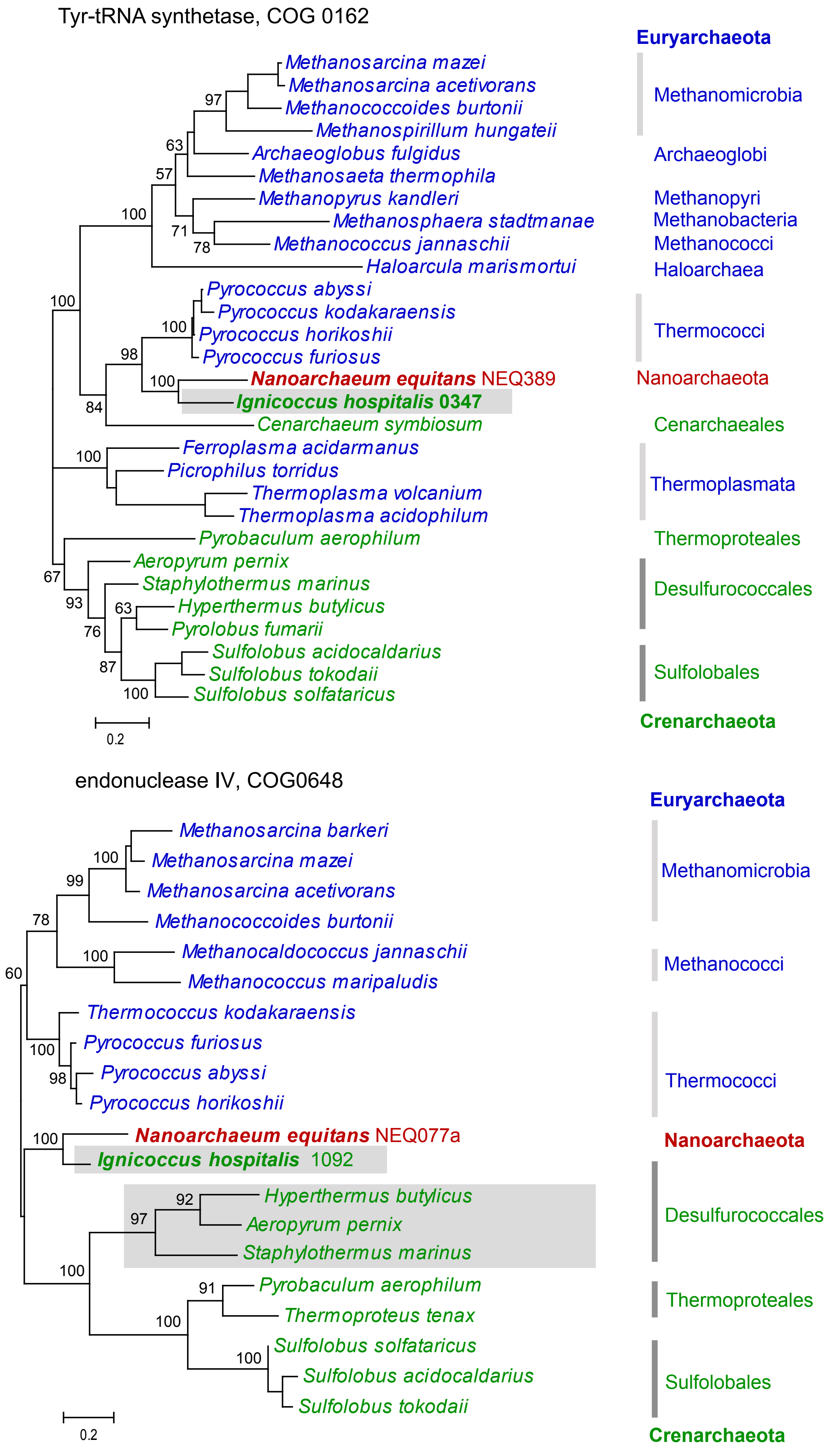

Figure S2. Maximum likelihood phylogenetic trees of archaeal tyrosyl-tRNA synthetases and of family IV endonucleases. Numbers indicate bootstrap support based on 100 replicates. Species that belong to the order Desulfurococcales are shaded. The scale bar indicates the inferred number of substitutions per site.

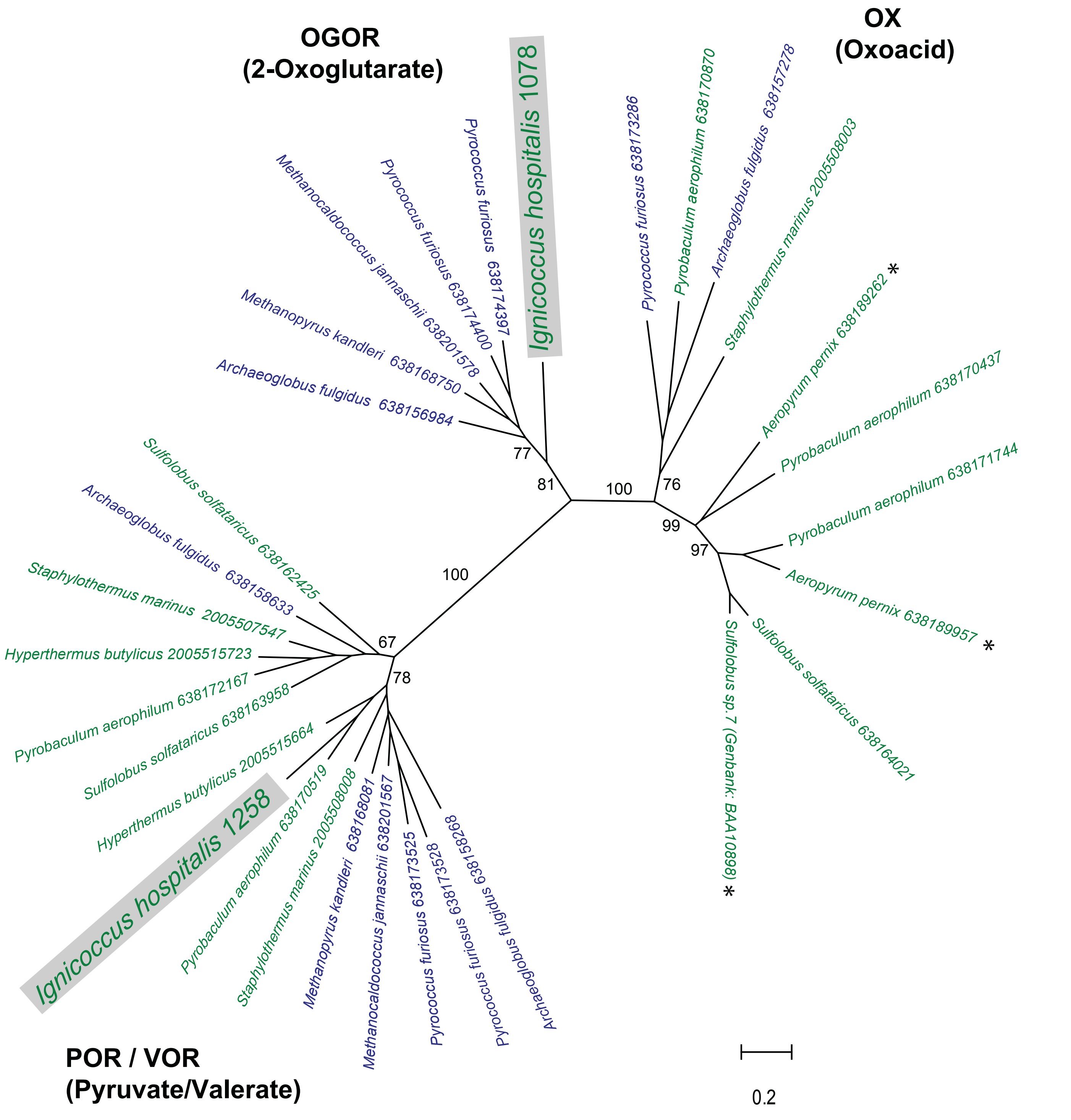

Figure S3. Maximum likelihood phylogenetic tree of the alpha subunit of archaeal 2-oxoacid: ferredoxin oxidoreductases. Green and blue indicates genes from species of the Crenarchaeota and Euryarchaeota, respectively and the gene ID values from the IMG database (http://img.jgi.doe.gov) are provided. The subfamilies specific for different 2-oxoacids are identified. Asterisks indicate enzymes that have been experimentally confirmed to accept multiple types of substrates. The numbers indicate bootstrap support based on 100 replicates and are shown only for the major clades. The scale bar indicates the inferred number of substitutions per site.

2-oxoacid:ferredoxin oxidoreductases

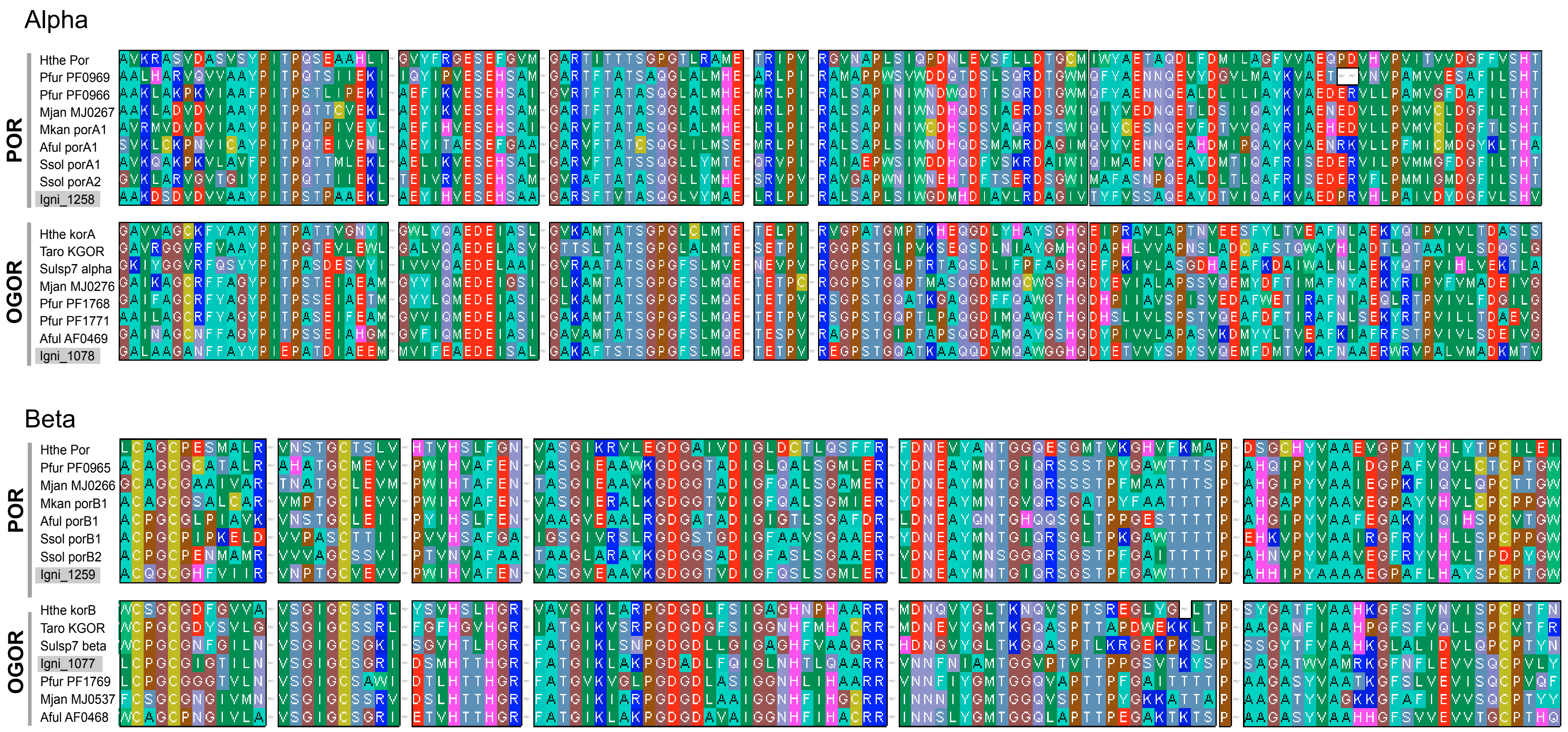

Figure S4. Amino acids-based sequence alignment of conserved regions of the alpha and beta subunits of pyruvate:ferredoxin oxidoreductases (POR) and 2-oxoglutarate:ferredoxin oxidoreductases (OGOR). The abbreviations correspond to species and gene names/ORF numbers (Bacteria: Hthe, *Hydrogenobacter thermophilus*; Taro, *Thauera aromatica*. Archaea: Pfur, *Pyrococcus furiosus*; Mjan, *Methanocaldococcus jannaschii*; Mkan, *Methanopyrus kandleri*; Aful, *Archaeoglobus fulgidus*; Ssol, *Sulfolobus solfataricus*, Sulsp7, *Sulfolobus* sp.7; Igni, *Ignicoccus hospitalis*).
